# Closed‐Loop Recyclable Silica‐Based Nanocomposites with Multifunctional Properties and Versatile Processability

**DOI:** 10.1002/advs.202304147

**Published:** 2023-10-16

**Authors:** Yi Hou, Guangda Zhu, Samantha O. Catt, Yuhan Yin, Jian Xu, Eva Blasco, Ning Zhao

**Affiliations:** ^1^ Beijing National Laboratory for Molecular Sciences Laboratory of Polymer Physics and Chemistry Institute of Chemistry Chinese Academy of Sciences Beijing 100190 P. R. China; ^2^ Institute for Molecular Systems Engineering and Advanced Materials (IMSEAM) Heidelberg University 69120 Heidelberg Germany; ^3^ Organic Chemistry Institute (OCI) Heidelberg University 69120 Heidelberg Germany

**Keywords:** chemical recycling, dynamic bonding, mechanical properties, organic–inorganic hybrid material

## Abstract

Most plastics originate from limited petroleum reserves and cannot be effectively recycled at the end of their life cycle, making them a significant threat to the environment and human health. Closed‐loop chemical recycling, by depolymerizing plastics into monomers that can be repolymerized, offers a promising solution for recycling otherwise wasted plastics. However, most current chemically recyclable polymers may only be prepared at the gram scale, and their depolymerization typically requires harsh conditions and high energy consumption. Herein, it reports less petroleum‐dependent closed‐loop recyclable silica‐based nanocomposites that can be prepared on a large scale and have a fully reversible polymerization/depolymerization capability at room temperature, based on catalysis of free aminopropyl groups with the assistance of diethylamine or ethylenediamine. The nanocomposites show glass‐like hardness yet plastic‐like light weight and toughness, exhibiting the highest specific mechanical strength superior even to common materials such as poly(methyl methacrylate), glass, and ZrO_2_ ceramic, as well as demonstrating multifunctionality such as anti‐fouling, low thermal conductivity, and flame retardancy. Meanwhile, these nanocomposites can be easily processed by various plastic‐like scalable manufacturing methods, such as compression molding and 3D printing. These nanocomposites are expected to provide an alternative to petroleum‐based plastics and contribute to a closed‐loop materials economy.

## Graphical abstract

Closed‐loop recyclable and less petroleum‐dependent silica‐based nanocomposites with glass‐like hardness, yet plastic‐like lightweight and toughness, as well as anti‐fouling, low thermal conductivity, and flame retardancy, combined with scalable plastic‐like manufacturing methods such as compression molding and 3D printing, promise to be an ideal alternative to plastics and contribute to a circular materials economy in a sustainable future.

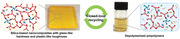

## 1 Introduction

Plastics have brought many conveniences to human life because of their flexibility, durability, light weight, and low cost.^[^ ^1^, ^2^, ^3^, ^4^ ^]^ However, about 40% of plastics are designed for short‐term use, and most of them (≈86%) are not recycled after disposal, becoming a great threat to the environment and human health.^[^ ^5^, ^6^, ^7^, ^8^ ^]^ The reuse of post‐consumer plastics is hampered by the deterioration of material performance and the high cost of sorting plastic waste.^[^ ^9^, ^10^, ^11^, ^12^ ^]^ In terms of circular economy, closed‐loop chemical recycling by depolymerizing plastics into monomers that can be repolymerized offers an alternative solution for retaining their original performance.^[^ ^13^, ^14^, ^15^, ^16^, ^17^, ^18^, ^19^, ^20^ ^]^ In this way, post‐consumer plastics are no longer waste, but raw feedstocks. Currently, closed‐loop chemically recyclable plastics have been reported, some of which are easy to prepare and recycle.^[^ ^21^, ^22^ ^]^ However, most plastics may originate from limited petroleum resources, and carbon, the primary element that makes up petroleum‐based plastics, is present in <0.03% of the earth's crust.^[^ ^11^ ^]^ Currently, the development of plastic substitutes such as paper and wood products can effectively alleviate the aggravation of so‐called “white pollution” to a certain extent, but these substitutes may lack desired properties such as flexibility, fire resistance, and waterproofing, etc., thus new alternatives are needed to fill this gap.^[^ ^23^, ^24^, ^25^, ^26^ ^]^ To achieve a sustainable future, high‐performance materials derived from abundant raw feedstocks that can be prepared on a large scale and recycled in a closed‐loop manner under mild conditions without causing secondary environmental pollution are ideal.^[^ ^27^ ^]^

**Figure 1:**
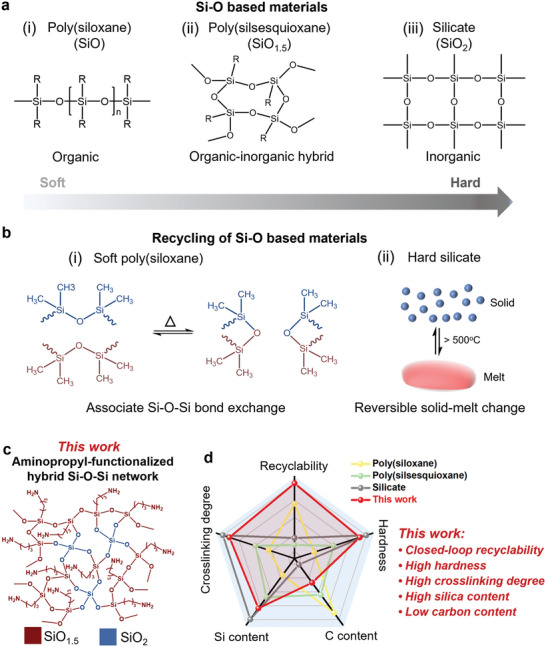
a) Classification of Si─O‐based materials, including poly(siloxane), poly(silsesquioxane), and silicate. b) Recycling mechanisms of Si─O‐based materials, including soft poly(siloxane), and hard silicate. c) Schematic diagram of the aminopropyl‐functionalized hybrid Si─O─Si network in this work. d) Comprehensive performance of our material in this work compared with common Si─O‐based materials.

Silicon occurs in nature as oxides and silicates and makes up ≈28% of the earth's crust. Although silicon is much more abundant than carbon‐based coal or crude oil (0.03%), it has a much smaller impact on our society compared to carbon. The main reason is that the Si─O bond is one of the strongest interactions in nature (bond energy of 534  kJ mol^−1^), making it very difficult to degrade by the reactions used for carbon‐based materials.^[^ ^28^ ^]^ The scope of Si─O‐based materials is vast, ranging from soft organic rubbers (e.g., poly(siloxane)) and organic–inorganic hybrid materials (e.g., poly(silsesquioxane)) to hard inorganic silicates (e.g., glass) (**Figure** **1**a). Organic poly(siloxane)s are quite soft while also being more petroleum‐dependent due to their low cross‐linked network consisting of SiO units and two carbon moieties in each unit (Figure 1a(i),d). So far, recycling of soft poly(siloxane)s has been achieved using the covalent network rearrangement based on an associative Si─O─Si bond exchange mechanism, in which the crosslinking density remains constant without depolymerization (Figure 1b(i),d).^[^ ^29^, ^30^, ^31^ ^]^ Non‐petroleum‐based inorganic silicates do not have any carbon, consist entirely of SiO_2_ units that are too hard, heavy, and fragile, and cannot be easily depolymerized under mild conditions due to the more highly cross‐linked networks than poly(siloxane)s and poly(silsesquioxane)s (Figure 1a(iii),d). For example, the recycling of glass requires a large amount of energy (>500 °C) to induce the transition from the solid to the flowable state (Figure 1b(ii)).^[^ ^32^, ^33^ ^]^ Organic–inorganic hybrid poly(silsesquioxane) with an intermediate cross‐linked network consisting of SiO_1.5_ units and only one carbon moiety in each unit shows less petroleum dependence, plastic‐like lightness and toughness, and glass‐like hardness, making it promising as a potential plastic substitute (Figure 1a(ii),d). However, closed‐loop recycling of Si─O‐based materials consisting of SiO_1.5_ units has not been achieved so far due to the lack of a dissociative reversible mechanism that allows the Si─O─Si network to fully depolymerize and repolymerize.

Previous reports have demonstrated that nucleophiles such as triethylamine (TEA) can accelerate the reversible hydrolysis and condensation reactions of small‐molecule aminopropyltriethoxysilanes (APTES) by blocking the H‐bonds between the aminopropyl and silanol groups, leaving the aminopropyl group in a free state to catalyze siloxane bond formation and hydrolysis.^[^ ^34^, ^35^, ^36^, ^37^ ^]^ However, we found that when moving to the polymer level, TEA could not completely depolymerize the aminopropyl‐modified poly(silsesquioxane) network consisting of SiO_1.5_ units (self‐condensed by APTES) with a high cross‐linking degree (94.8%). In this work, the stronger nucleophiles diethylamine (DEA) and ethylenediamine (EDA) are used because they can more effectively block H‐bonds between silanol and aminopropyl, leaving more aminopropyl in the free state, resulting in closed‐loop recycling of hybrid Si─O─Si network consisting of SiO_1.5_ and SiO_2_ units at room temperature, even though its cross‐linking degree (97.0%) is higher than the aminopropyl‐modified poly(silsesquioxane) (Figure 1c,d).

Based on this reversible hybrid Si─O─Si network, we report for the first time the closed‐loop recycling of less petroleum‐dependent silica‐based nanocomposites with higher Si content and lower C content than poly(silsesquioxane) (Figure 1c,d). The reversible polymerization/depolymerization of aminopropyl‐functionalized hybrid Si─O─Si networks is catalyzed by more efficient free aminopropyl groups from commercial monomers with highly nucleophilic DEA and EDA. Notably, rigid Si─O─Si networks with high cross‐linking degree are prone to fracture due to the initial stresses formed during the drying process, and water needs to be avoided during service as it can trigger depolymerization. By introducing soft polymeric micelles and fluorinated compounds into the network, intact and waterproof nanocomposites are successfully achieved. These nanocomposites show excellent glass‐like transparency and high hardness (Figure 1d), yet plastic‐like light weight and toughness, exhibiting the highest specific mechanical strength superior to that of common materials such as PMMA, glass, and ZrO_2_ ceramic, as well as multifunctionality, i.e., anti‐fouling, low thermal conductivity, and flame retardancy. The migration of fluorinated compounds facilitates the formation of an inherently hydrophobic protective layer at the outermost of the nanocomposite, imparting good water resistance and chemical durability in daily use. When the protective layer is disrupted, the nanocomposites can be chemically depolymerized by water alone under ambient conditions without the use of strong acids, bases, or organic solvents. Moreover, selective removal of the nanocomposites from polymer wastes is easy and possible. In addition, the flowability of the prepolymer solution and the water‐induced plasticity of the nanocomposites enable a variety of simple plastic‐like processing techniques, including sol–gel–solid processing, compression molding, and 3D printing. Such commercial and cost‐effective feedstocks, easy processing, and environmentally friendly depolymerization procedures facilitate large‐scale production and closed‐loop recycling. We envision a promising and exciting time when such silica‐based nanocomposites can replace some petroleum‐based plastics and contribute to a more sustainable future.

## 2 Results and Discussion

### 2.1 Closed‐Loop Recyclable Si─O─Si Networks

**Figure 2:**
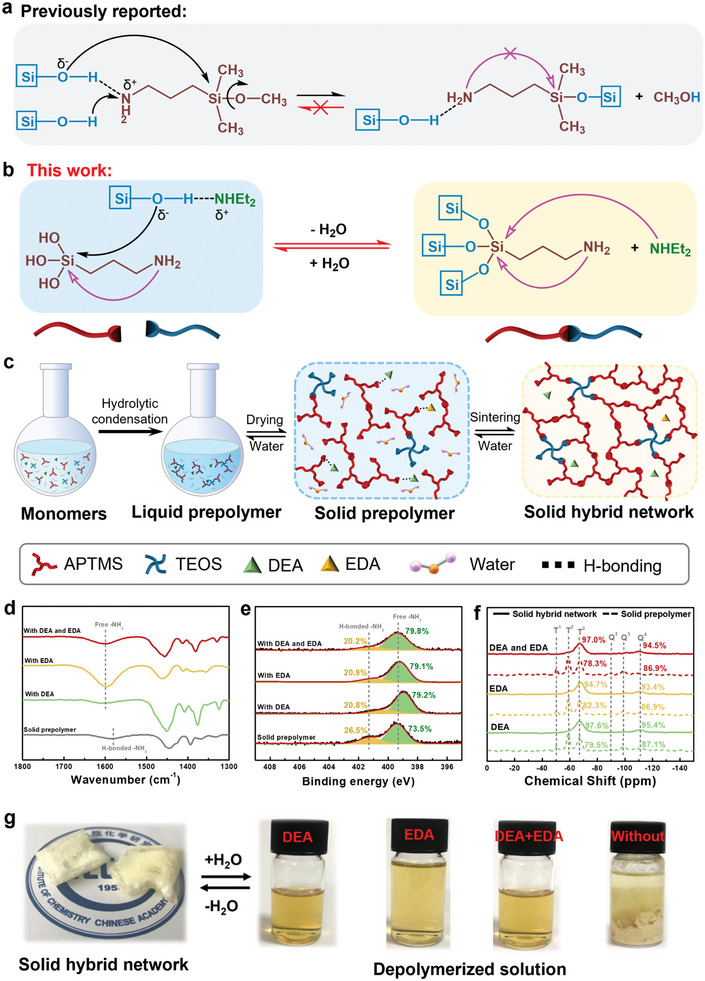
Dynamic Si─O─Si bonds and the reversible depolymerization and repolymerization of hybrid Si‐O‐Si networks. a) The previously reported reaction of the aminosilane with Si─OH group to form Si─O─Si bond at room temperature. b) Illustration of reversible Si─O─Si and Si─OH bonds using the catalyst of DEA (NHEt_2_) in this work. c) Illustration of the preparation process of the hybrid Si─O─Si networks. d) FTIR spectra of the solid prepolymers. e) The high‐resolution N1s peaks in XPS curves of the solid prepolymers. f) ^29^Si MAS NMR spectra and condensation degrees of solid prepolymers and solid hybrid networks. g) Photos of the resultant hybrid material after sintering at 300 °C for 3 h and the depolymerized solutions with/without DEA or EDA.

As discussed previously, aminosilanes, such as APTES and aminopropyltrimethoxysilane (APTMS), show the unique capability of reversible hydrolysis and condensation due to the catalytic effect of aminopropyl groups. At room temperature, each aminosilane can occupy two silanol sites: one condensed with another Si─OH group to form a Si─O─i bond, and the second H‐bonded to the aminopropyl group (**Figure** **2**a). The H‐bonded aminopropyl groups are less reactive and unable to exert a catalytic effect, meaning they cannot nucleophilically attack the silicon atom in the presence of water and therefore cannot hydrolyze the formed Si─O─Si bonds to Si─OH (Figure 2a). However, the introduction of nucleophiles such as TEA, DEA, or EDA enables the reversibility between the formed Si─O─Si bond and Si─OH (Figure 2b). Because TEA, DEA, and EDA are stronger bases than the aminopropyl group, they can replace it to form hydrogen bonds with Si─OH, leaving aminopropyl groups in the free state. Subsequently, the free aminopropyl groups and TEA, DEA, or EDA can nucleophilically attack the silicon atom. Therefore, in the presence of water, the TEA, DEA, or EDA can facilitate the formation of hydrogen bonding with Si─OH and further the conversion of Si─O─Si to Si─OH; in the absence of water, the Si─OH is catalyzed to form Si─O─Si bonds (Figure 2b). Nevertheless, following our previously reported strategy,^[^ ^38^ ^]^ this network consisting of SiO_1.5_ units could only be partially depolymerized in water in the presence of TEA, meaning that it could not be recycled in a closed‐loop manner (Figure S1, Supporting Information).

In order to improve the system, we have developed a much harder aminopropyl‐functionalized hybrid Si─O─Si network (hardness >1.0 GPa) in the presence of DEA and EDA enabling reversible depolymerization and repolymerization at room temperature (as shown in schematically in Figure 2c). The hybrid Si─O─Si networks were prepared by the co‐condensation of commercial APTMS and tetraethylorthosilicate (TEOS) monomers with the catalyst of DEA and EDA, followed by drying and sintering (Figure 2c, Figure S2a‐c, Supporting Information). The resultant hybrid networks consisted of SiO_1.5_ (from APTMS) and SiO_2_ (from TEOS) units with a high degree of condensation (DC) (DC_APTMS_ = 97.0%, DC_TEOS_ = 94.5%, Table S1, Supporting Information), which endowed the final silica‐based nanocomposites with a higher hardness (hardness > 1.0 GPa, discussed in detail later). As mentioned, DEA and EDA are more nucleophilic than TEA, so they can form strong hydrogen bonds with silanol more effectively and block the hydrogen bonds between silanol and aminopropyl. Fourier transform infrared (FTIR) spectra of the prepolymers show a N─H bending mode at 1578 cm^−1^, indicating that the ─NH_2_ in aminopropyl groups are H‐bonded to silanols. After the incorporation with DEA or EDA, bands appeared at ≈1600 cm^−1^ due to the bending mode of free ─NH_2_ (Figure 2d). X‐ray photoelectron spectroscopy (XPS) results also indicate that the ratio of free aminopropyl groups to H‐bonded aminopropyl groups increased after the incorporation of DEA or EDA (Figure 2e). In the presence of water, the free aminopropyl groups can attack the silicon atoms (Figure 2b), leading to the rapid hydrolysis of the hybrid Si─O─Si network (as shown schematically in Figure 2c). It should be noted that the drying and sintering processes lead to the volatilization of DEA and EDA from prepolymer sol, but the resulting highly cross‐linked Si─O─Si network can lock up a portion of the DEA and EDA. In addition, the hydrogen bonding between DEA or EDA and silanols also makes them less likely to escape. The residual DEA and EDA are sufficient to induce free aminopropyl groups even after two recycling cycles. (Figure S3, Supporting Information).

More importantly, the free aminopropyl groups of APTMS catalyze the hydrolysis of the Si─O─Si network more effectively than that of the APTES oligomers, which can act synergistically with stronger nucleophilic DEA or EDA and allow the hybrid Si─O─Si networks, even with a DC up to 97.6%, to be quickly depolymerized in water under ambient conditions. The solid‐state^29^Si magic‐angle spinning (MAS) nuclear magnetic resonance (NMR) spectroscopy results indicate that the ratios of T^1^ (R‐Si(OSi)(OH)_2_) and T^2^ (R‐Si(OSi)_2_(OH)) to T^3^ (R‐Si(OSi)_3_) (T^1^+T^2^+T^3^ = 1) and Q^1^ (Si(OSi)_1_(OH)_3_), Q^2^ (Si(OSi)_2_(OH)_2_) and Q^3^ (Si(OSi)_3_(OH)_1_) to Q^4^ (Si(OSi)_4_) (Q^1^+Q^2^+Q^3^+Q^4^ = 1) decrease significantly when the samples are in contact with water for a certain time, indicating the breaking of the Si─O─Si network and the formation of silanols (Figure 2f, Table S1, Supporting Information). A clear solution of depolymerized products with DEA or EDA can be observed (Figure 2g). In contrast, strong hydrogen bonds are formed between silanol and aminopropyl groups without DEA or EDA (as shown schematically in Figure S4, Supporting Information). The H‐bonded aminopropyl groups are less reactive to attack silicon atoms, especially in rigid Si─O─Si networks with a high DC. In the control experiment, the hybrid Si─O─Si network without the additional DEA or EDA cannot be depolymerized, even after being immersed in water for several months (Figure 2g). The extensive depolymerization of the hybrid Si─O─Si network was demonstrated by matrix‐assisted laser desorption/ionization‐time of flight mass spectrometry (MALDI‐TOF‐MS). The depolymerized products have a molecular weight varying from ≈400 to 700 g mol^−1^ (Figure S5, Supporting Information), indicating that the highly cross‐linked Si─O─Si network recovers to the prepolymers. The presumptions of possible chemical structural units of the prepolymers and the depolymerized products corresponding to the main peaks in the MALDI‐TOF‐MS spectra (Figure S5, Supporting Information) are shown in Tables S2 and S3 (Supporting Information). The depolymerized products consist mainly of two or three SiO_1.5_ units with/without one H‐bonded DEA and EDA or a SiO_2_ unit, similar to the prepolymers. While TEOS plays a role in increasing the cross‐linking degree and improving the strength of the hybrid Si─O─Si network, as the content of TEOS is low (≈15 wt.%), the irreversible Si─O─Si network self‐condensed by TEOS rarely occurs in hybrid networks, thus showing little effect on the depolymerization. MALDI‐TOF‐MS spectra of the poly(silsesquioxane) network formed by the self‐condensation of APTMS show similar results to those of hybrid networks (molecular weights ranging from 400 to 700), further demonstrating that TEOS has little effect on depolymerization (Figure S6, Supporting Information). The ^1^H‐NMR and ^13^C‐NMR spectra prove that the chemical structures of the depolymerized products are consistent with those of the original prepolymers (Figures S7 and S8, Supporting Information). By drying the depolymerized products and then sintering them following the same procedure used previously, the Si─O─Si network could be reconstructed, demonstrating the closed‐loop recycling capability.

### 2.2 Design and Properties of Silica‐Based Nanocomposites

**Figure 3:**
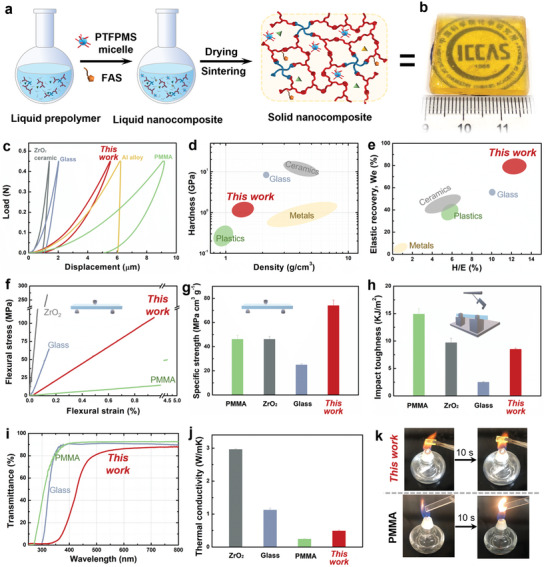
Material design and properties. a) Preparation process of the nanocomposite. b) Photos of the resultant nanocomposite. c) Load‒displacement curves from the indentation of our nanocomposite and common materials. d) Comparison of the hardness and density of the nanocomposites with common materials. e) Comparison of the calculated hardness‐to‐Young's modulus (*H/E*) ratio and elastic recovery rate (*W_e_*) of the nanocomposites with common materials. f) Flexural stress‒strain curves and g) specific strength. h) Charpy impact toughness of the nanocomposite and common materials. i) Optical transmittance of glass, PMMA, and the nanocomposite. j) Thermal conductivity of the nanocomposite and common materials. k) Photographs of the nanocomposite and PMMA on the alcohol lamp.

In a next step, nanocomposites based on the reversible Si─O─Si network were designed and prepared for practical applications. Although we have achieved the closed‐loop recycling of the hybrid Si─O─Si network, it should be noted that the processing of bulk materials with extremely rigid networks is usually not as easy as for soft polymeric materials, and several challenges still need to be addressed. First, the necessary procedures of drying the prepolymer solution into solid material and further sintering help to increase the cross‐linking degree to obtain super hard bulk materials by the condensation reaction. However, our hybrid Si─O─Si networks were easily damaged by the initial stress and prone to fracture during the aforementioned process (Figure S9a‐c, Supporting Information). Second, contact with water needs to be avoided during the service life of our materials, as this may trigger depolymerization. To that end, soft nanofillers (poly[(3,3,3‐trifluoropropyl) methylsiloxane] (PTFPMS)@APTES micelles) and a low surface tension compound (1H, 1H, 2H, 2H‐perfluorodecyltriethoxysilane, FAS) were incorporated into the network (**Figure** **3**a). The successful introduction of fluorinated components of PTFPMS and FAS into the nanocomposites can be demonstrated by the F elements measured by XPS and SEM EDS (Figure S10, Supporting Information). The APTES shell of PTFPMS@APTES micelles and FAS could be cross‐linked with the hybrid Si─O─Si network (Figure S2d‐f, Supporting Information), so that the micelle nanofillers and FAS could be uniformly distributed in a continuous matrix, resulting in regular and uniform arrangement of soft PTFPMS@APTES micelles and FAS as fillers and rigid hybrid Si─O─Si network as a continuous matrix. The uniform soft micelles effectively dissipate the initial stress during the drying and further sintering process of the prepolymer solution. An intact and transparent bulk nanocomposite without cracks could be obtained (Figure 3b; Figure S9d, Supporting Information). The fluorinated components migrate from uniformly crosslinked FAS and PTFPMS@APTES micelles in matrix and form uniform low surface energy nanodomains on the outermost surface of nanocomposite, as evidenced by the homogeneously dispersed F elements in the SEM EDS (Figure S10c, Supporting Information), imparting excellent water repellency to the surface.

Next, the influence of the catalyst on the mechanical properties and decomposition rate of the nanocomposites was carefully investigated. We found that DEA resulted in a higher DC (DC_APTMS_ = 97.6%, DC_TEOS_ = 95.4%) than EDA (DC_APTMS_ = 94.7%, DC_TEOS_ = 93.4%) (Figure 2f; Table S1, Supporting Information). As a result, the nanocomposite with DEA shows higher hardness and flexural strength than the nanocomposite with EDA (Figure S11, Supporting Information). In turn, the depolymerization rate of the nanocomposite with DEA is slower than that with EDA (Figure S12, Supporting Information). To obtain a compromise of hardness, flexural strength, and depolymerization rate, the nanocomposite with DEA and with EDA were mixed at a volume ratio of 3:1 to prepare the nanocomposite with DEA and EDA, which shows similar closed‐loop recycling characteristics (Figures S5–S8 and S12 and Tables S2 and S3, Supporting Information).

The combination of soft micelles and a rigid Si─O─Si network contributes to the superior mechanical properties of the nanocomposite. First, the surface mechanical properties by nanoindentation were evaluated. The nanoindentation curves (Figure 3c) show that the nanocomposite exhibits the smallest curve hysteresis compared to Al alloy, poly(methyl methacrylate) (PMMA), glass, and ZrO_2_ ceramic, revealing a much better surface elastic deformability. The highly cross‐linked network endows our material with a hardness (*H*) of 1.02±0.07 GPa, much higher than that of most plastics (*H*<0.3 GPa).^[^ ^39^ ^]^ The hardness is comparable to that of some metals, but with a density of approximately 1.36 g cm^‐3^, is as light as some plastics (Figure 3d). Materials with high hardness and low Young's modulus (*E*), satisfying *H/E*≥10% and elastic recovery rate *W_e_* ≥60%, exhibit comprehensive properties of being hard, flexible, and resilient.^[^ ^25^, ^38^, ^40^, ^41^ ^]^ The hybrid Si─O─Si network provides high hardness, while the soft micelles decrease the modulus (*E* = 8.44±0.41 GPa), resulting in an *H/E* of 12.1±0.4% and *W_e_* of 79.8±0.4%, superior to typical plastics, metals, and ceramics (Figure 3e; Table S4, Supporting Information). In general, hardness, and flexibility are mutually exclusive.^[^ ^43^, ^44^, ^45^, ^46^, ^47^ ^]^ The simultaneous high hardness and flexibility of our nanocomposite makes it suitable as a scratch‐tolerant material compared to glass; that is, the surface shows no visible scratches from a glass cutter, whereas the brittle nature of glass makes its surface susceptible to forming scratches under the same friction (Figure S13, Supporting Information). The nanocomposite also exhibits exceptional mechanical properties in bulk. Generally, glass or ceramic materials have extremely high hardness and strength but lack substantial toughness. Our nanocomposite is less brittle than glass and ZrO_2_ ceramic while also exhibiting both great toughness and flexural deformability (Figure 3f). With a low density of 1.36 g cm^‐3^, the nanocomposite exhibits the highest specific strength (75.0 MPa cm^3^ g^‐1^) of ZrO_2_ ceramic, glass, and PMMA (Figure 3g). This high specific strength and toughness endow this material with excellent impact resistance. The Charpy impact test yields an impact resistance of 8.5 KJ m^‐2^, which is better than glass and comparable to ZrO_2_ ceramic (Figure 3h). These low density, high hardness and toughness, high specific strength, and excellent impact‐proof and scratch‐resistant properties may make this nanocomposite ideal for engineering applications, for example in fields such as aerospace, intelligent automobiles, and green building. Integrating a hydrophobic protective layer that can migrate repeatedly and improving the mechanical properties of the nanocomposites may greatly reduce the possibility for external damage and any further water‐induced depolymerization, thereby increasing the stability of the nanocomposites under usual service.

Other functionalities of this nanocomposite, such as high transparency, low thermal conductivity, and flame retardancy, inspire greater potential for engineering applications than conventional materials. The uniform distribution of nanosized micelles in the continuous silica matrix contributes to an average transmittance of 88% at visible light wavelengths, which is notably comparable to the excellent transparency of glass and PMMA (≈90%) (Figure 3i). Interestingly, the nanocomposite shows absorption of ultraviolet (UV) light due to its light‐yellow color. In comparison with ZrO_2_ ceramic and glass, the nanocomposite exhibits a significantly low thermal conductivity and can serve as a thermally insulating material (Figure 3j). The unique optical performance and low thermal conductivity make our material a desirable candidate for energy‐efficient buildings, where it could prevent excessive UV radiation indoors and provide better insulation than concrete, brick, stone, and steel. Moreover, it shows no visible change up to 400 °C in both nitrogen and air, demonstrating much better thermostability than most widely used polymers. The theoretical mass percentage of inorganic units (SiO_1.5_ and SiO_2_) in the resultant nanocomposites is calculated to be ≈52wt.%, thus over 60wt.% of the residual at 700 °C (Figure S14, Supporting Information) supports that our material may mainly composed of inorganic units, showing great potential to reduce the utilization of petroleum‐based feedstocks and with a low carbon footprint. Moreover, the abundant silica components and amino groups contribute to excellent fire retardancy. No flammability could be observed even when the material was ignited for 60 s, in contrast to PMMA, which burned within 10 s (Figure 3k). Our nanocomposites are the first to combine light weight, high strength, and toughness, thermal insulation, and fire retardancy properties all in one transparent material.

### 2.3 Closed‐Loop Recycling of Silica‐Based Nanocomposites

**Figure 4:**
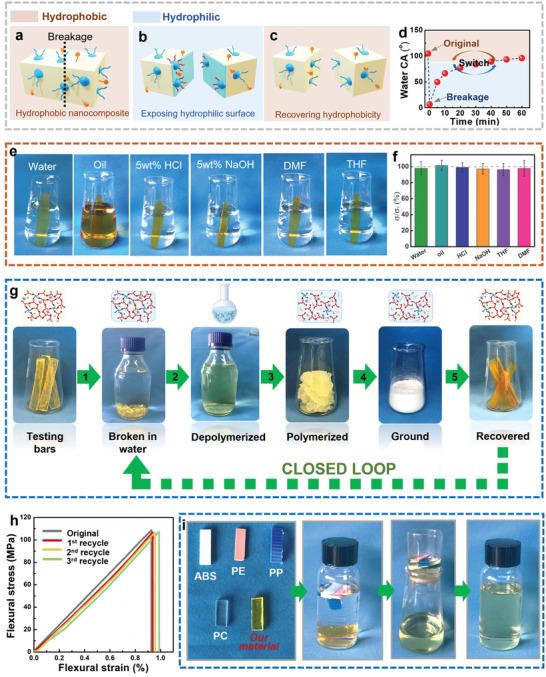
Closed‐loop recycling of the nanocomposite. Schematic illustration of the nanocomposite with a) the original hydrophobic surface and the fractured surface switching from (b) hydrophilic to (c) hydrophobic. d) The change in the contact angle of the freshly fractured surface with time. e) Photographs of the nanocomposites in various solvents after 24 h. f) The retention rate of the flexural strength of the nanocomposites after immersion in various solvents for 24 h. g) The closed‐loop recycling process: the testing bars of the original nanocomposites, the freshly broken bars exposing hydrophilic surfaces in water, the depolymerized products in water, the regenerated products after drying, the ground powder, and the recycled testing bars. h) Mechanical properties of the original and recycled nanocomposites. i) The selective recycling of our nanocomposite from a plastic waste stream (acrylonitrile butadiene styrene plastic (ABS), polyethylene (PE), polypropylene (PP) and polycarbonate (PC)).

The depolymerization of this nanocomposite depends on the interaction of the reversible Si─O─Si network with water and can therefore be controlled by switching the hydrophilicity and hydrophobicity. The spontaneous surface enrichment of the low‐surface‐energy fluorinated components of PTFPMS and FAS makes the outermost layer of the nanocomposite repellent to water, with an original water contact angle (CA) of 104±2° (**Figure** **4**a,d). After the nanocomposite is broken with force, the freshly exposed fractured surface is mainly dominated by the Si─O─Si network that is hydrophilic with a minimum CA of 7±2° (Figure 4b,d). The fluorinated components gradually migrate to the fractured surface to recover hydrophobicity (CA >90° after 50 min of air exposure) (Figure 4c,d). However, the fractured hydrophilic surface can absorb water to induce the depolymerization of the Si─O─Si network before the hydrophobicity is recovered. The transformation between hydrophilic and hydrophobic character is therefore used as a “switch” for on‐demand recycling.

When undamaged, the surface layer of the nanocomposite has sufficiently robust resistance to water, and aqueous solutions of HCl and NaOH (5 wt.%), as well as organic solvents (tetrahydrofuran, *N,N*‐dimethylformamide) and olive oil (Figure 4e). The flexural strengths and CAs of the nanocomposites did not change significantly after 24 h of immersion in these liquids (Figure 4f, Figure S15, Supporting Information). Moreover, acrylic paint sprayed on the surface could be easily wiped off even after 24 h of drying, which was not the same case for glass (Figure S16, Supporting Information). This anti‐fouling property could eliminate the traditional cleaning steps required prior to recycling, thereby saving costs and improving efficiency. The inherent protective outer layer inhibits water‐induced depolymerization of the nanocomposite materials, endowing them with excellent water impermeability and chemical durability throughout daily use.

Closed‐loop recycling of the nanocomposite was realized by exposing the hydrophilic Si─O─Si network to water, as demonstrated by recycling 20.0 g of testing bars in 500 mL of deionized water (Figure 4g). A clear solution was obtained by depolymerizing the freshly broken bars in water at room temperature for 10 h. The recycled aqueous solution can be dried, sintered, and then ground into powder with a weight recovery of 96%. The recycled solid prepolymer powder can be used to reprocess the testing bars. The mechanical properties of the reprocessed materials from the recycled solutions changed minimally even after three recycling loops (Figure 4h), and the recycling ratio of both flexural strength and strain at break of the nanocomposite can reach ≈100% (Figure S17, Supporting Information). Dynamic light scattering and transmission electron microscopy (TEM) results showed that the micelles were dispersed uniformly in the solution of depolymerized products (Figure S18, Supporting Information). A shorter depolymerization time could be achieved by increasing the recycling temperature (Figure S19, Supporting Information). Generally, the recycling of plastics is limited by the high cost and time‐consuming process of sorting plastic waste streams.^[^ ^9^, ^10^, ^11^, ^12^ ^]^ It is notable that the depolymerization of our material could be achieved at room temperature by using water alone. The closed‐loop recycling under mild conditions is therefore cost‐effective and convenient, suggesting suitability for industrial‐scale applications without secondary environmental pollution. Figure 4i demonstrates that for our material, plastic waste sorting is unnecessary, as the material can be easily depolymerized in water and then separated from common plastics through a simple filtration process.

### 2.4 Diverse Processing Methods for Silica‐Based Nanocomposites

**Figure 5:**
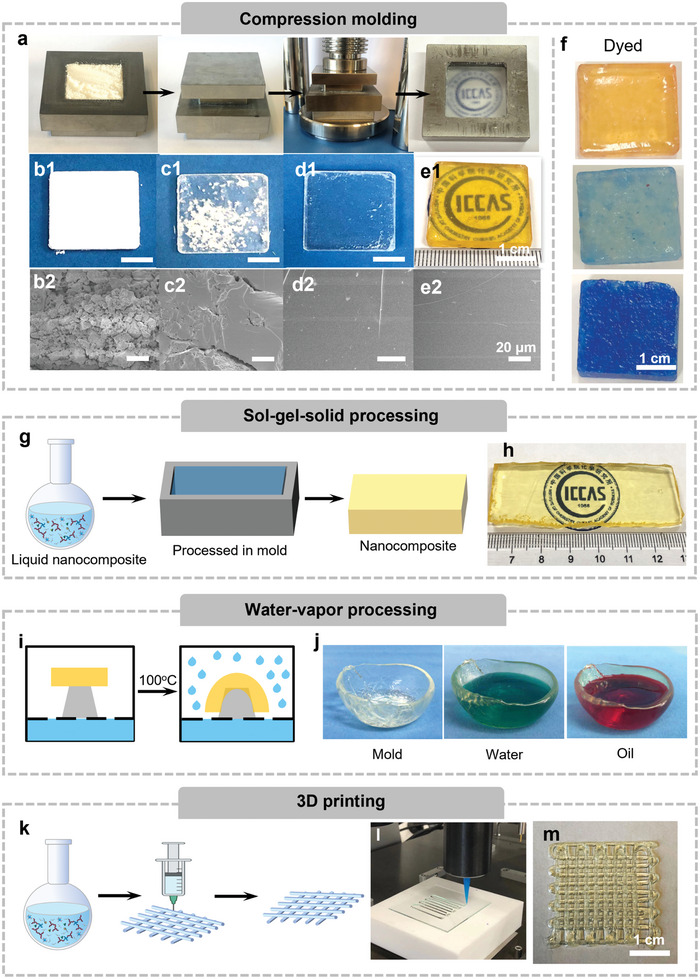
Processing methods of the nanocomposite. a–f) Compression molding: a) Photographs of the compression molding process and the resultant samples. b–e) Photographs and cross‐sectional SEM images of the samples prepared under 5 MPa (b1‐b2), 10 MPa (c1‐c2), 15 MPa (d1‐d2), and sintered nanocomposites (e1‐e2). f) Photos of the colored nanocomposites by adding different amounts of CuCl_2_. g,h) Sol–gel–solid processing: g) the schematic illustration of sol–gel–solid processing, and h) photo of the obtained nanocomposite. i,j) Water‐vapor processing: i) schematic illustration of water‐vapor processing, and j) photos of the obtained cups for liquids. k–m) 3D printing: k) schematic illustration of 3D printing, photos of (l) 3D printing process and (m) the printed structure.

In addition to the superior mechanical strength and multifunctionality exhibited by these closed‐loop recyclable materials, such as light weight, excellent glass‐like hardness, plastic‐like toughness, transparency, thermal insulation, anti‐fouling, and fire retardancy properties, etc, their simple processability is even more critical for practical applications. Even though the resultant nanocomposites are extremely hard, the flowability of the liquid prepolymer and the water‐induced plasticity of the nanocomposites enable various plastic‐like processing techniques, as demonstrated in **Figure** **5**. When considering scaling up production to commercial levels, compression molding processing offers a time‐ and energy‐effective way to directly process the recovered powder into bulk nanocomposite materials with intentional or predesigned size and shape within several minutes under mild conditions (Figure 5a). The powder (with a water content of ≈15 wt.%, Figure S20, Supporting Information) could be fused together within 5 min via the reconstruction of the Si─O─Si network under different pressures at ambient temperature (Figure 5b1,d1). A transition from white porous to transparent dense bulks was achieved by increasing pressure from 5 to 15 MPa, indicating a pressure‐dependent fusion degree (Figure 5b2,d2). After further sintering, the intact and transparent nanocomposite could be obtained (Figure 5e1,e2). Although the drying and sintering processes involve specialized equipment, multiple steps, and long time, most of the processes are well‐established and widely used in the processing of rigid inorganic silica‐based materials. In addition, all water‐soluble molecules, or water‐dispersible nanomaterials, such as metal ions or nanoparticles, can be incorporated into the nanocomposites, providing different appearances and properties. For example, light or dark blue colors could be obtained by adding different concentrations of CuCl_2_ (Figure 5f). To further demonstrate the feasibility of this material in industrial applications, it was shown that the incorporation of a large amount of cheaper fumed SiO_2_ nanoparticles into the nanocomposites reduces the amount of prepolymer solution used, thereby reducing the cost of feedstock in large‐scale preparations (Figure S21, Supporting Information). Typically, the incorporation of excessive rigid nanofillers causes discontinuous internal structures, making materials brittle, and poorly fracture‐resistant. However, our compression molding processing allows the preparation of intact nanocomposites, even with the addition of 25 and 28 wt.% SiO_2_ nanofillers, while retaining high mechanical strength, low thermal conductivity, and high thermal stability (Figure S22, Supporting Information). Meanwhile, the introduction of CuCl_2_ and fumed SiO_2_ nanoparticles into the nanocomposites do not affect the stability of the nanocomposites in water, as evidenced by similar surface and mechanical properties before and after immersion in water (Figure S23, Supporting Information). For sol‐gel‐solid processing, the prepolymer solution was cast into the mold and then dried at 60 °C for 24 h (Figure 5g), and transparent nanocomposites could be obtained after drying and sintering (Figure 5h).

In addition to the above‐mentioned 2D structures, more challenging 3D structures and morphologies could also be achieved. Taking advantage of the water‐induced plasticity, the nanocomposite plate could be softened by water or water vapor and then reshaped to form free‐standing cups (Figure 5i,j). The inherent amphiphobicity forming the outermost layer facilitates resistance to long‐term erosion by water or oil (Figure 5j), making the structures stable for daily use. This is a demonstration of a mechanically demanding everyday plastic item that requires recyclability after its limited lifespan. Moreover, 3D printing has become the gold standard in fabricating complex 3D architectures.^[^ ^46^, ^47^, ^48^, ^49^, ^50^ ^]^ It is also particularly desirable in closed‐loop recycling where the high‐performance properties of the materials are fully retained. We show extrusion‐based 3D printing of the liquid prepolymer from the viscoelastic liquid state, its fluid‐like behavior allows the fabrication of a lattice structure with multiple layers (Figure 5k–m).

## 3 Conclusion

In summary, silicon is much more abundant in the earth's crust than carbon, and organic–inorganic hybrid poly(silsesquioxane)s consisting of SiO_1.5_ units with only one carbon moiety in each unit shows less petroleum dependence and excellent mechanical strength, demonstrating promise as potential plastic substitutes. However, reversible depolymerization and repolymerization of poly(silsesquioxane)s consisting of SiO_1.5_ units have not been achieved until now. Herein, we have presented a simple and scalable strategy to fabricate a closed‐loop recyclable silica‐based nanocomposite based on an aminopropyl‐functionalized Si─O─Si network consisting of SiO_1.5_ and SiO_2_ units co‐condensed from commercial monomers of APTMS and TEOS. The hybrid Si─O─Si network with DC up to 97.0% can be depolymerized and then repolymerized by efficient catalytic effects of free aminopropyl groups, with the assistance of highly nucleophilic DEA and EDA. Water‐induced circular recyclability allows the nanocomposite to be processed and recycled on a large scale under mild conditions while avoiding secondary environmental pollution. The structural design of soft PTFPMS@APTES micelles and FAS as fillers and rigid hybrid Si─O─Si network as a continuous matrix enables, for the first time, the integration of multiple superior properties in one fully recyclable nanocomposite. The nanocomposites show plastic‐like light weight and toughness, glass‐like transparency, and hardness, in combination with multifunction such as anti‐fouling, water resistance, low thermal conductivity, and fire retardancy. Furthermore, scalable plastic‐like manufacturing methods, such as compression molding and 3D printing, facilitate widespread practical application while reducing plastic pollution and fossil energy depletion for a closed‐loop material economy.

## 4 Experimental Section

### Materials

3‐Aminopropyltrimethoxysilane (APTMS, 97%), 3‐aminopropyltriethoxysilane (APTES, 97%), and tetraethylorthosilicate (TEOS, 98%) were purchased from J&K Scientific Co., Ltd. Diethylamine (DEA, >99%) was purchased from Aladdin Co., Ltd. Ethylenediamine (EDA, >99%) was purchased from Xilong Scientific Co., Ltd. Poly[(3,3,3‐trifluoropropyl) methyl siloxane] (PTFPMS) (M_w_ = 1.06×10^5^, PDI = 1.4) was supplied by Chengdu Tuoli Co., Ltd. Triethylamine (TEA) (>99%) and 1H, 1H, 2H, 2H‐perfluorodecyltriethoxysilane (FAS) were purchased from Sigma–Aldrich. Fumed silica (SiO_2_) was purchased from Shanghai Maklin Biochemical Co., Ltd. Tetrahydrofuran (THF, 99%), *N,N*‐dimethylformamide (DMF, 99.9%), hydrochloric acid (HCl, 36–38% aqueous solution), sodium hydroxide (NaOH, 96%), ethanol (95%) and ethyl acetate (>99.5%) were purchased from Beijing Chemical Works. Press‐type water‐based acrylic paint spray and olive oil were obtained through commercial purchase. All the reagents were used without further purification. Commercially available poly(methyl methacrylate) (PMMA), polycarbonate (PC), acrylonitrile butadiene styrene plastic (ABS), polypropylene (PP), polyethylene (PE), glass, zirconium dioxide (ZrO_2_), aluminum oxide (Al_2_O_3_), copper and aluminum alloy were used.

### Material Preparation

Preparation of the hybrid silica‐based materials from APTMS and TEOS containing DEA: APTMS (60 mL) and TEOS (15 mL) were dissolved in a mixture of 16 mL water and 11 mL ethanol, and then 168 µL HCl was added. Prepolymer sol was obtained by stirring the mixture at 80 °C under 1000 r.p.m. for 24 h. Then, 10 mL of DEA was added dropwise to the prepolymer sol and stirred at 80 °C for another 24 h. Finally, the prepolymer sol containing DEA was dried at 80 °C for 24 h, and then sintered under nitrogen protection according to the following procedure: at a heating rate of 0.8°C min^−1^ to 100 °C and holding for 2 h, to 200 °C and holding for 2 h, and then to 300 °C and holding for 3 h. In the control experiment, removing TEOS from the recipe and repeating the above steps resulted in silica‐based materials containing DEA from the self‐condensation of APTMS.

Preparation of the hybrid silica‐based materials from APTMS and TEOS containing EDA: 10 mL of EDA was used instead of DEA and other compositions and procedures were the same as the above nanocomposite containing DEA. In the control experiment, silica‐based materials containing EDA were obtained from the self‐condensation of APTMS.

Preparation of PTFPMS@APTES micelle dispersion: PTFPMS (2.5 g) was dissolved in 50 mL ethyl acetate, and then 50 mL ethanol was added dropwise under stirring to induce the formation of PTFPMS micelles. APTES (5 mL) was added to the above dispersion under stirring and stewed at 80 °C for 2 days. Then a stable dispersion of PTFPMS@APTES micelles was obtained.

Preparation of the nanocomposites containing DEA and EDA: 3 µL of FAS and 30 mL of PTFPMS@APTES dispersion were added into the prepolymer sols containing DEA and EDA, respectively, and then stirred at 80 °C and 1000 rpm for 24 h. The above mixtures were mixed at a volume ratio of 3:1, and then dried into wet powders with a water content of ≈15 wt.%. Then, the wet powders were compression molded at 15 MPa. After drying the nanocomposites at 80 °C for 3 days, the samples were sintered following the same procedure mentioned above. In the control experiments, nanocomposites were also prepared by a similar method using prepolymer sols containing DEA and EDA.

Preparation of poly(silsesquioxane) with TEA: APTES (30 mL) was dissolved in a mixture of 8 mL water and 8 mL ethanol, and then 84 µL HCl was added to induce the hydrolysis and condensation of APTES. Poly(silsesquioxane) sol was obtained by stirring the mixture at 80 °C and 1000 rpm for 24 h. Then, TEA was added, and then the mixture was stirred at 80 °C and 1000 rpm for 24 h (the fraction of TEA in the mixture was 4.0 wt%). After drying the mixture at 80 °C for 3 days, it was sintered according to the procedure in the preparation of the nanocomposite containing DEA or EDA.

3D printing of the lattice structure: Lattice structures were prepared by a bioplotter pneumatic dispensing system (BioScaffolder 2.1, GeSiM, Grossermannsdorf, Germany). The nanocomposite sols were plotted on a glass slide through a conical needle at room temperature under a certain pressure. After printing, the 3D lattice structures were removed from the glass side and then dried and sintered as above.

### Mechanical Testing

The nanoindentation test was conducted using an Agilent Nano Indenter G200 system equipped with a diamond Berkovich tip, and the experiments were performed based on continuous stiffness measurement (CSM). For each force loading, holding, and withdrawing cycle, the maximum load was 450 mN, the loading and unloading rates were both 20 mN s^−1^ and the holding time at the maximum load was 0.5 s. The nanoindentation hardness and effective Young's modulus were calculated using the Oliver‐Pharr method. The elastic recovery (W_e_) was calculated using areas under the loading and unloading curves. Three‐point bending tests and puncture tests were carried out on a SUNS UTM4104 Instrument at room temperature (≈26 °C). For three‐point bending tests, samples with sizes of 70 mm × 10 mm × 3 mm were used. The tests were performed at a loading rate of 1.0 mm min^−1^ with a support span of 48 mm. Charpy impact tests of the samples were performed on a Chengde Bao Hui XJJY‐5 pendulum impact tester, and the dimensions of the tested samples were 70 mm × 10 mm × 3 mm. Each kind of sample was tested at least five times.

### Characterization

Fourier transform infrared (FTIR) spectra were recorded on a Bruker Tensor27 FTIR spectrophotometer by 32 scans from 4000 to 600 cm^−1^, with a resolution of 4 cm^−1^. The samples were prepared by casting the dispersions on KBr pellets. X‐ray photoelectron spectroscopy (XPS) measurements were carried out on a Thermo Scientific ESCALab250Xi using 200 W monochromatic Al Kα radiation. Solid‐state ^29^Si MAS NMR (magic angle spinning nuclear magnetic resonance) investigation was performed on Avance Ш 400, Bruker production. The degree of condensation (DC) of APTMS was calculated from the peak areas of T^1^ (R‐Si(OSi)(OH)_2_), T^2^ (R‐Si(OSi)_2_(OH)) and T^3^ (R‐Si(OSi)_3_) according to the following equation: DC = (T^1^+2T^2^+3T^3^)/3. The degree of condensation of TEOS was calculated from the peak areas of Q^1^ (R‐Si(OSi)(OH)_3_), Q^2^ (R‐Si(OSi)_2_(OH)_2_), Q^3^ (R‐Si(OSi)_3_(OH)) and Q^4^ (R‐Si(OSi)_4_) according to the following equation: DC = (Q^1^+2Q^2^+3Q^3^+4Q^4^)/4. Autoflex III MALDI‐TOF‐MS was used to characterize the molecular weight of the samples with water as the solvent. Nuclear magnetic resonance (NMR) spectra were recorded on a Bruker Advance III 500 MHz spectrometer using D_2_O as the solvent. The thermal conductivities were measured at 25 °C using Tci (C‐THERM, Canada). Thermogravimetric analysis (TGA) was carried out on a TA Instruments Q600 Simultaneous Thermal Analyzer. Samples were heated in a nitrogen atmosphere at a rate of 10 °C min^−1^ from room temperature to 700 °C. Transmittance spectra were obtained by using a UV‒vis spectrometer (Lambda 950, Perkin Elmer), and the average transmittance in the range of 400–800 nm was calculated. The contact angle (CA) and sliding angle (SA) were measured using a KRÜSS DSA 100 by a sessile drop method. Water and kerosene were used for CA (5 µL) and SA (10 µL) measurements, and each value was an average of three tests.

## Conflict of Interest

The authors declare no conflict of interest.

## Supporting information

Supporting Information

## Data Availability

The data that support the findings of this study are available in the supplementary material of this article.
