# Supplementary material for: Closed‐Loop Recyclable Silica‐Based Nanocomposites with Multifunctional Properties and Versatile Processability
Source: Adv Sci (Weinh). 2023 Oct 16;10(35):2304147. doi: 10.1002/advs.202304147 (PMC10724396; doi:10.1002/advs.202304147)
Supplement: Supplementary file 1 — Supporting Information [file ADVS-10-2304147-s001.pdf]

ADVANCED SCIENCE
Open Access

Supporting Information

for *Adv. Sci.*, DOI 10.1002/advs.202304147

Closed-Loop Recyclable Silica-Based Nanocomposites with Multifunctional Properties and Versatile Processability

Yi Hou, Guangda Zhu, Samantha O. Catt, Yuhan Yin, Jian Xu, Eva Blasco and Ning Zhao**

Supporting Information

Closed-Loop Recyclable Silica-Based Nanocomposites with Multifunctional Properties and Versatile Processability

Yi Hou, Guangda Zhu, Samantha O. Catt, Yuhan Yin, Jian Xu, Eva Blasco, and Ning Zhao**

*Corresponding author. Email: eva.blasco@oci.uni-heidelberg.de; zhaoning@iccas.ac.cn

Supplementary Figures

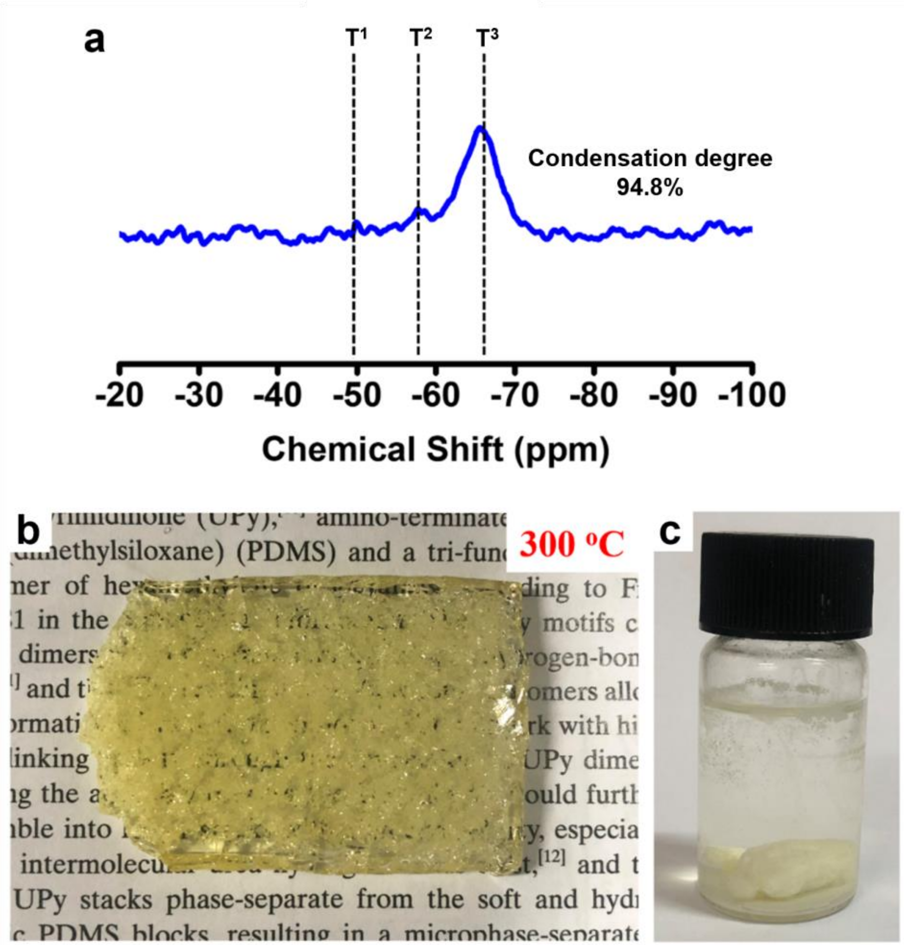

Figure S1. Poly(silsesquioxane) network prepared by the self-condensation of APTES with TEA as the catalyst. (a) ^{29}Si MAS NMR spectrum and condensation degree of the sintered poly(silsesquioxane) network. (b) Photograph of the resultant bulk material after sintering at 300°C. (c) Photograph of the sintered poly(silsesquioxane) in water, demonstrating that the network could not be depolymerized completely.

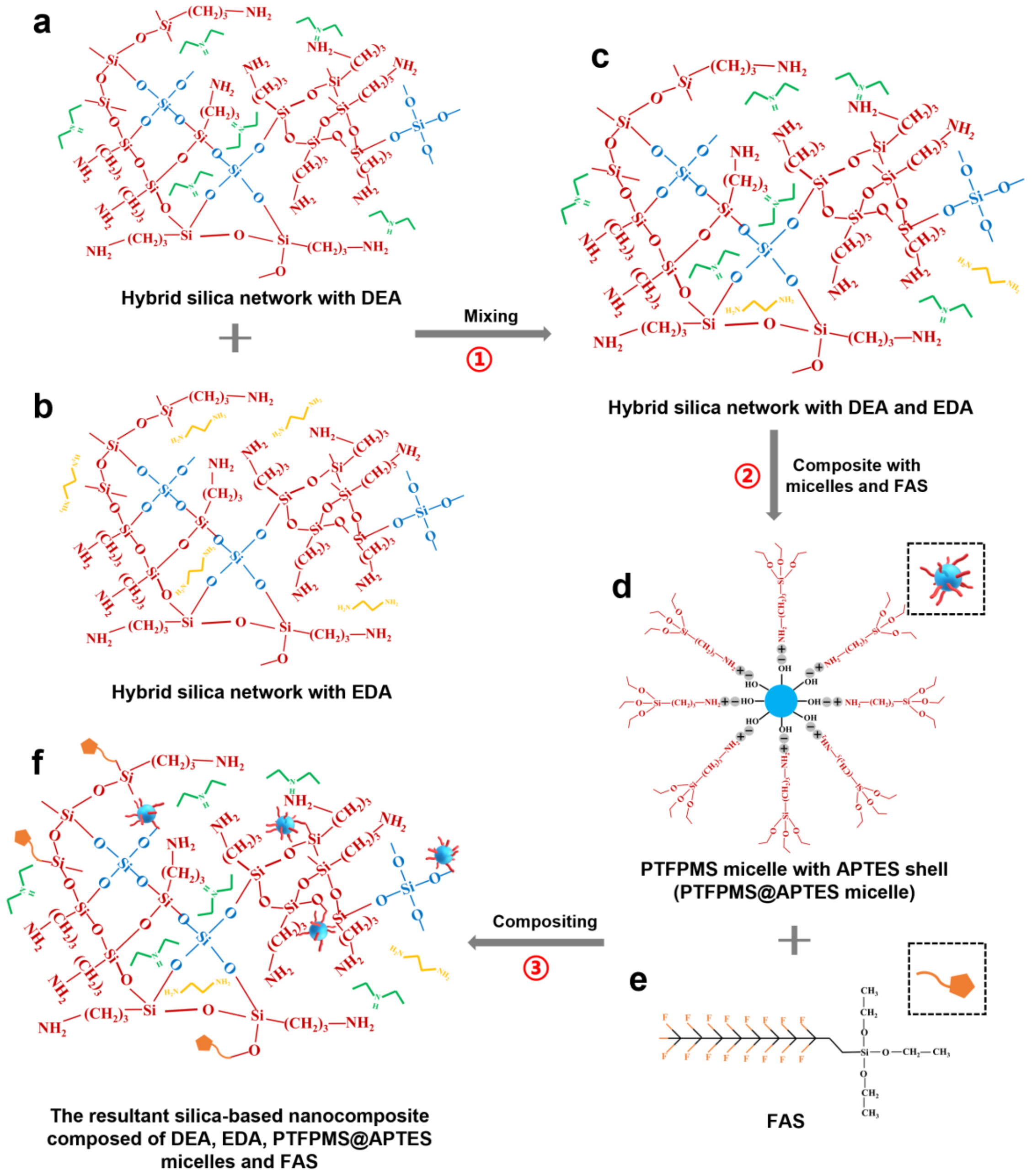

Figure S2. Schematic chemical structures of the closed-loop recycled nanocomposite. The hybrid Si-O-Si networks with (a) DEA, (b) EDA, and (c) DEA and EDA. (f) The resulting silica-based nanocomposites containing (d) PTFPMS micelles with an APTES shell and (e) FAS.

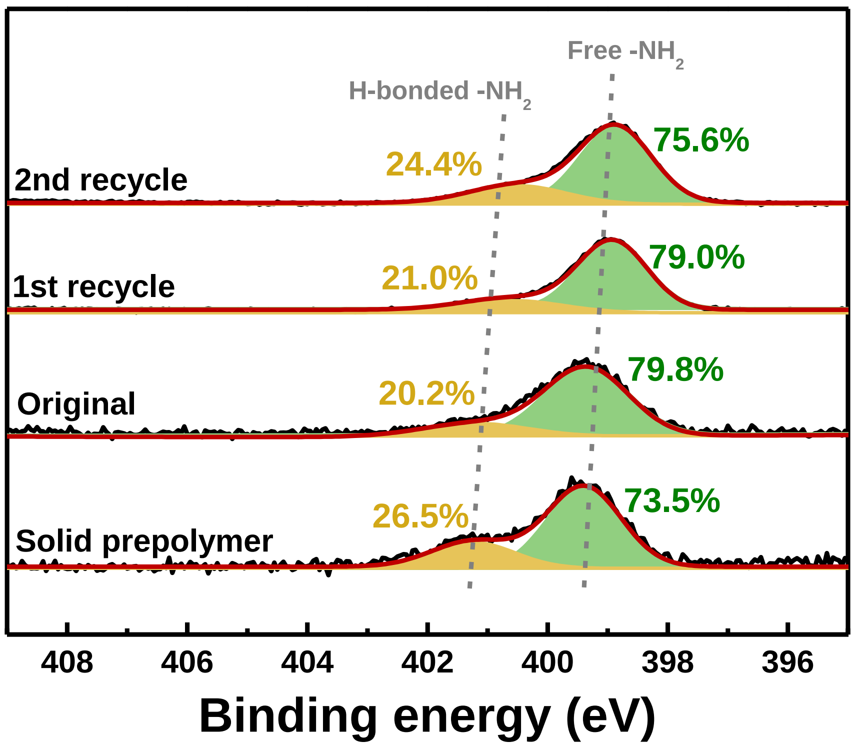

Figure S3. The high resolution N1s peaks in XPS curves of solid prepolymer, the original and recycled solid prepolymers with DEA and EDA.

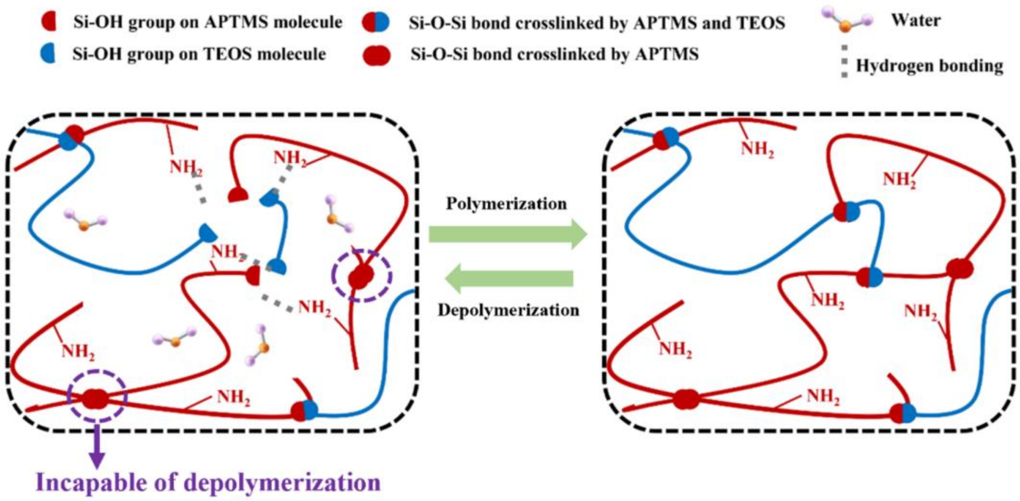

Figure S4. Schematic illustration of the partial depolymerization of the hybrid Si-O-Si network without a catalyst. Strong hydrogen bonds are formed between silanols and aminopropyl groups in the network without a catalyst. The hydrogen-bonded aminopropyl groups are less reactive, leading to the hybrid Si-O-Si network being only partially depolymerized.

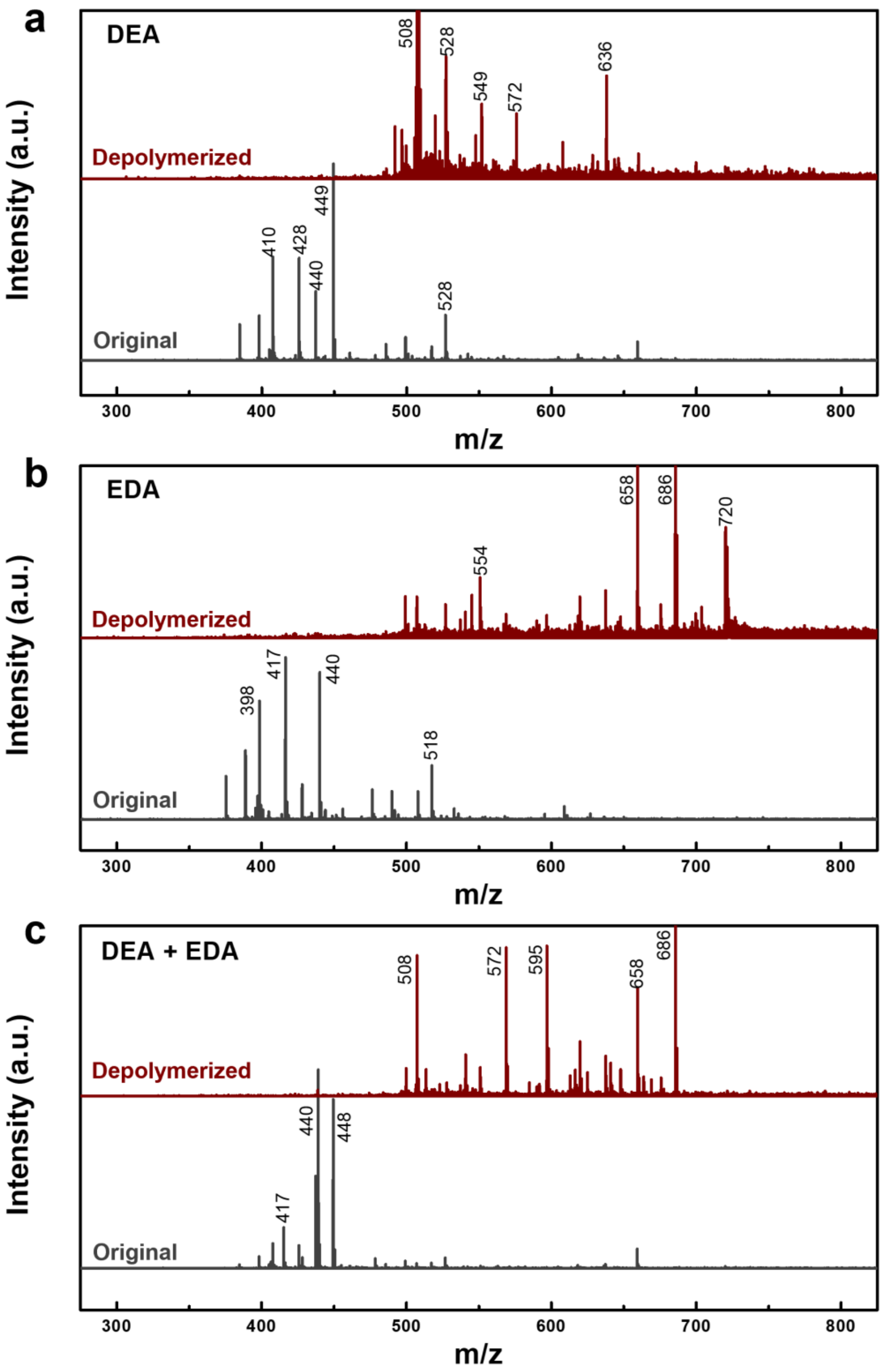

Figure S5. The MALDI-TOF-MS spectra of the prepolymers and the depolymerized products of the hybrid Si-O-Si networks formed from the co-condensation of APTMS and TEOS with the catalyst: (a) DEA, (b) EDA, and (c) DEA and EDA.

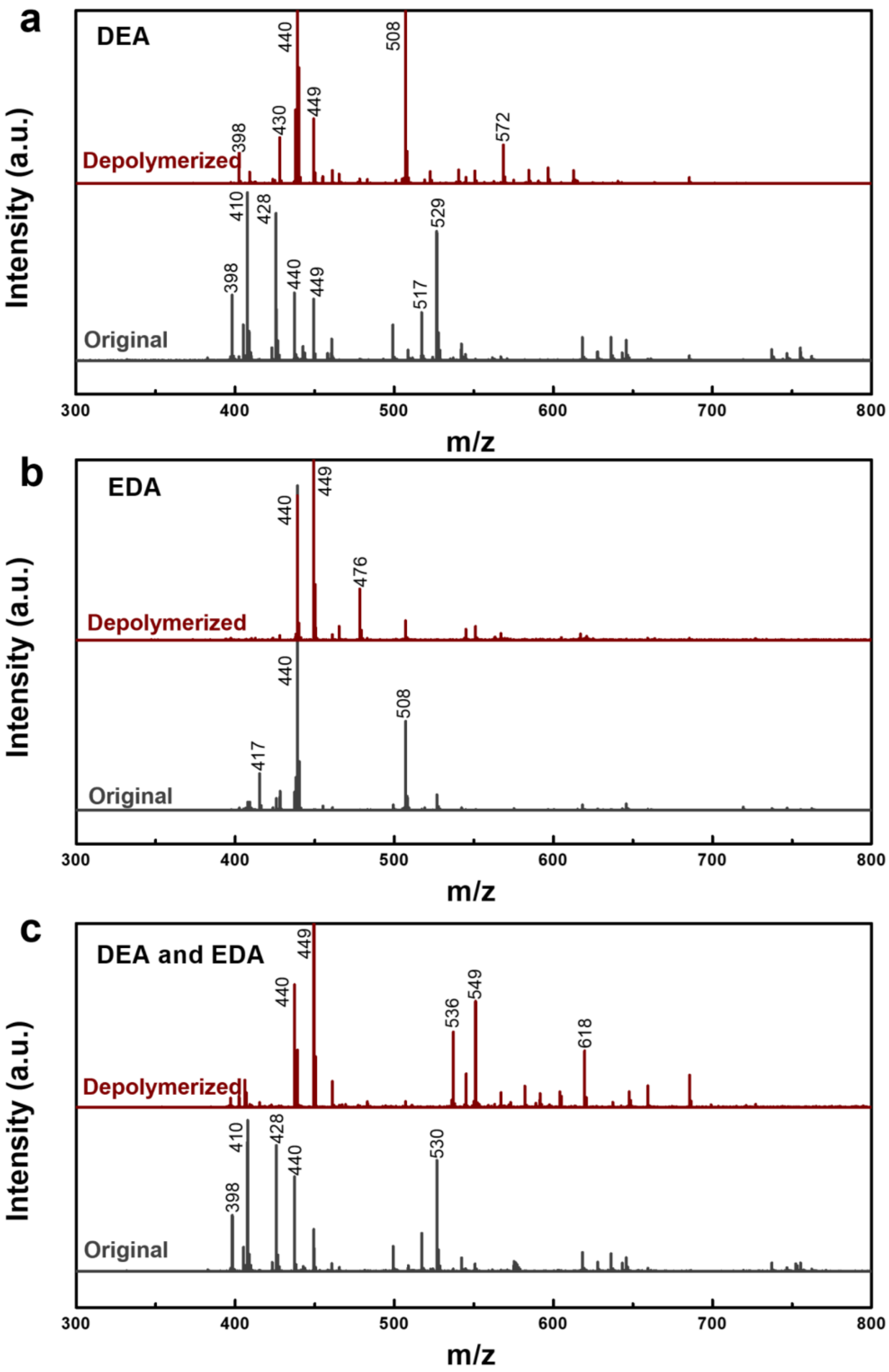

Figure S6. The MALDI-TOF-MS spectra of the prepolymers and the depolymerized products of the poly(silsesquioxane) networks formed from the self-condensation of APTMS with the catalyst: (a) DEA, (b) EDA, and (c) DEA and EDA.

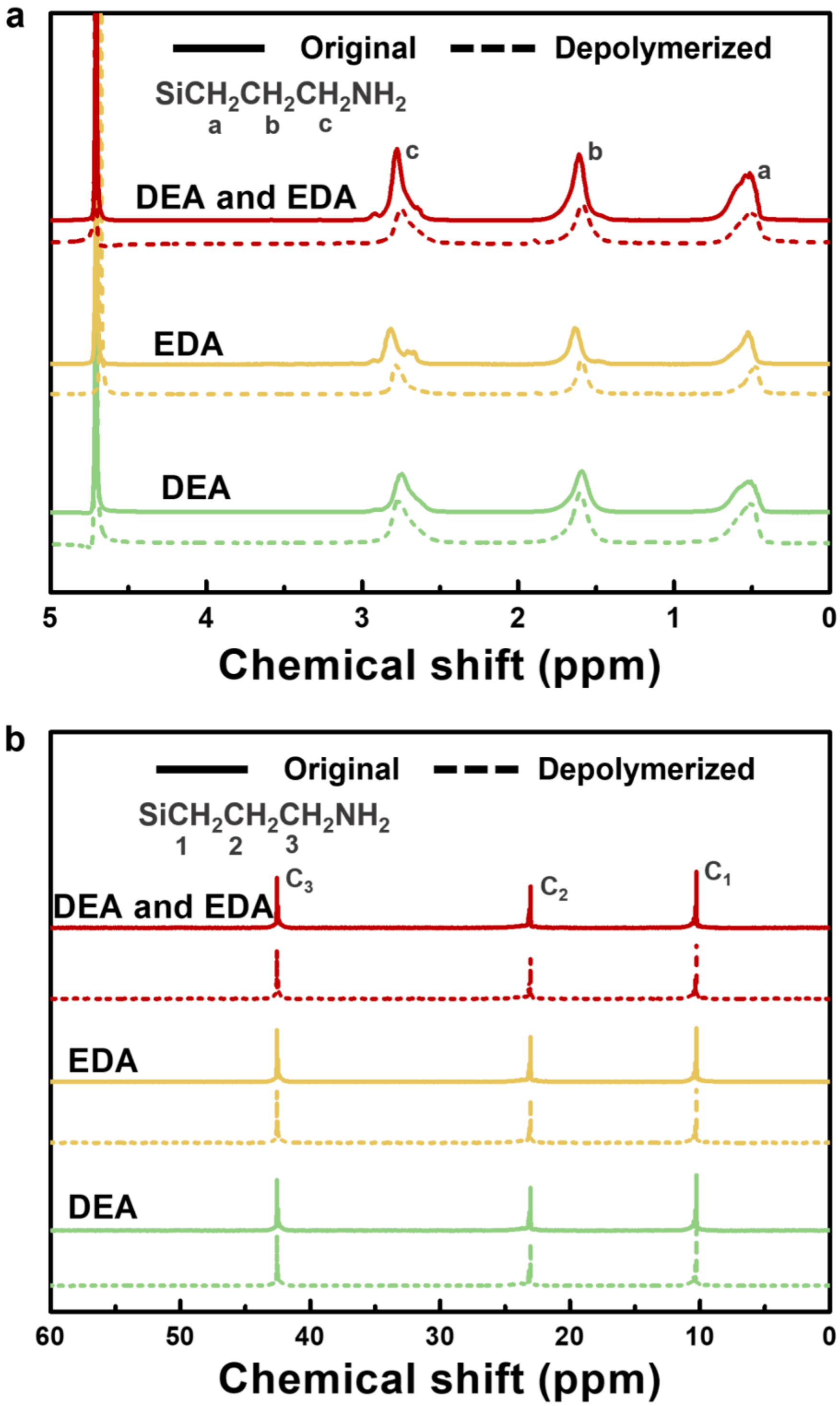

Figure S7. The depolymerization of the hybrid Si-O-Si networks formed from the co-condensation of APTMS and TEOS with DEA, EDA and the mixture of DEA and EDA: (a) ^{1}H-NMR spectra and (b) ^{13}C-NMR spectra of the corresponding prepolymers and the depolymerized products.

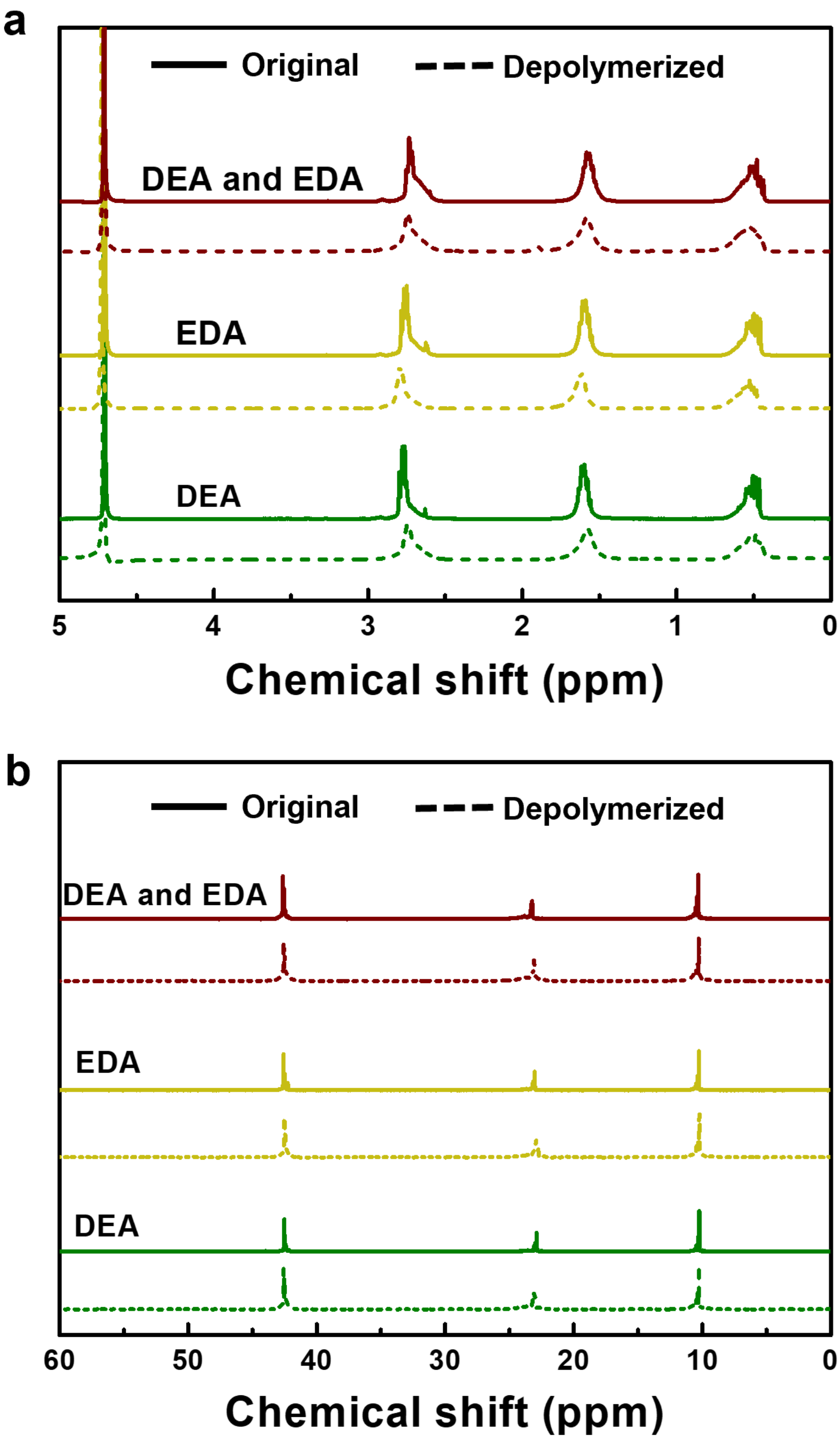

Figure S8. The depolymerization of the poly(silsesquioxane) networks formed from the self-condensation of APTMS with DEA, EDA and the mixture of DEA and EDA: (a) ^{1}H-NMR spectra and (b) ^{13}C-NMR spectra of the corresponding prepolymers and the depolymerized products.

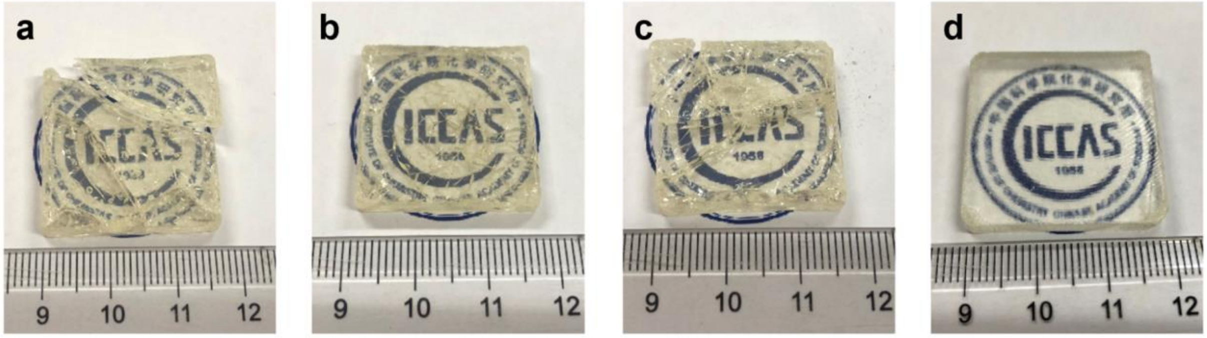

Figure S9. Effect of the micelles on the formation of defect-free bulk materials after drying. Photographs showing that without micelles, the materials containing (a) DEA, (b) EDA, and (c) DEA and EDA, cracked after drying at 80°C for 3 days. (d) Photograph of the intact nanocomposite containing DEA and EDA with micelles after drying at 80°C for 3 days.

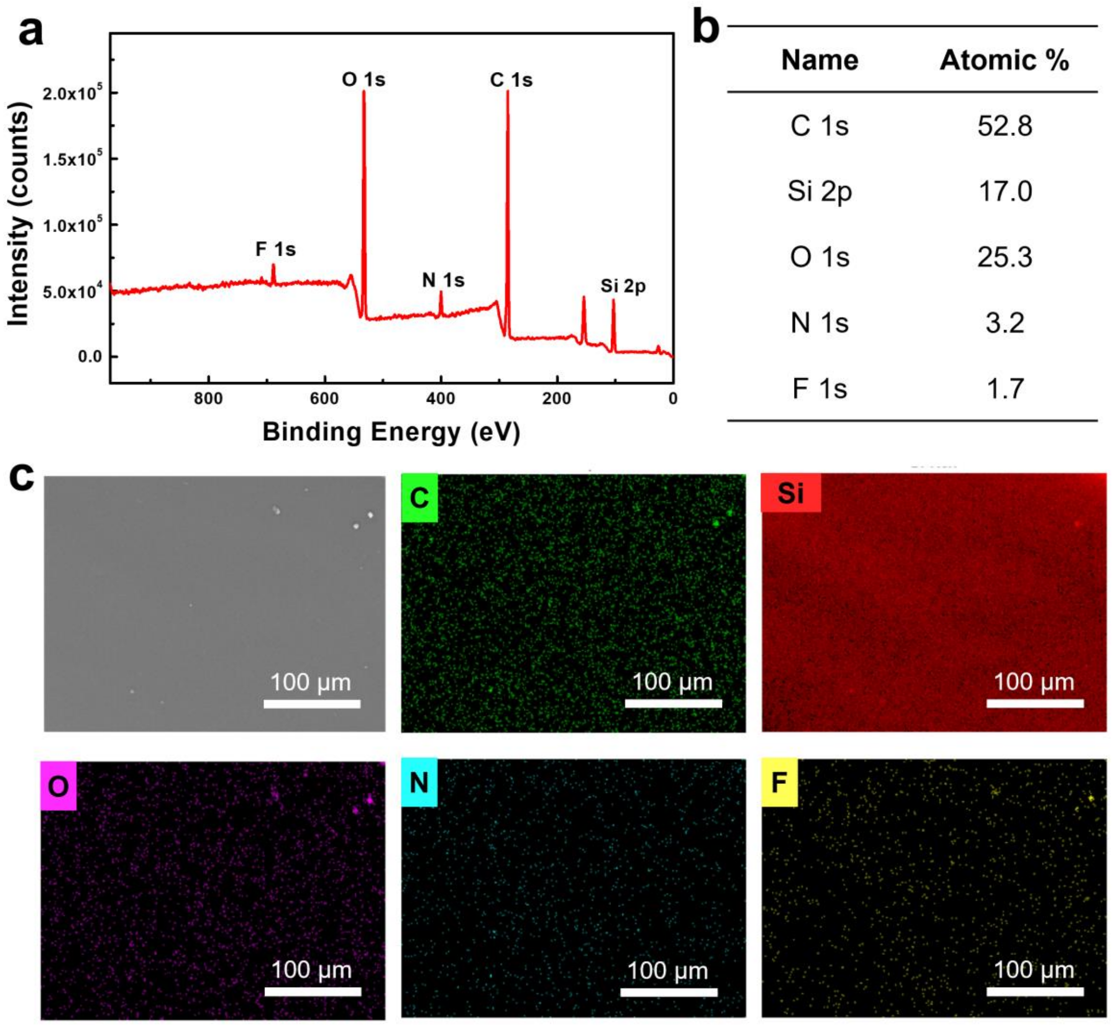

Name	Atomic %
C 1s	52.8
Si 2p	17.0
O 1s	25.3
N 1s	3.2
F 1s	1.7

Figure S10. Elemental analysis of the nanocomposite: (a) XPS spectrum, (b) quantification table indicating the atomic species and their atomic percentages; (c) SEM image and corresponding element mapping of the fracture surface.

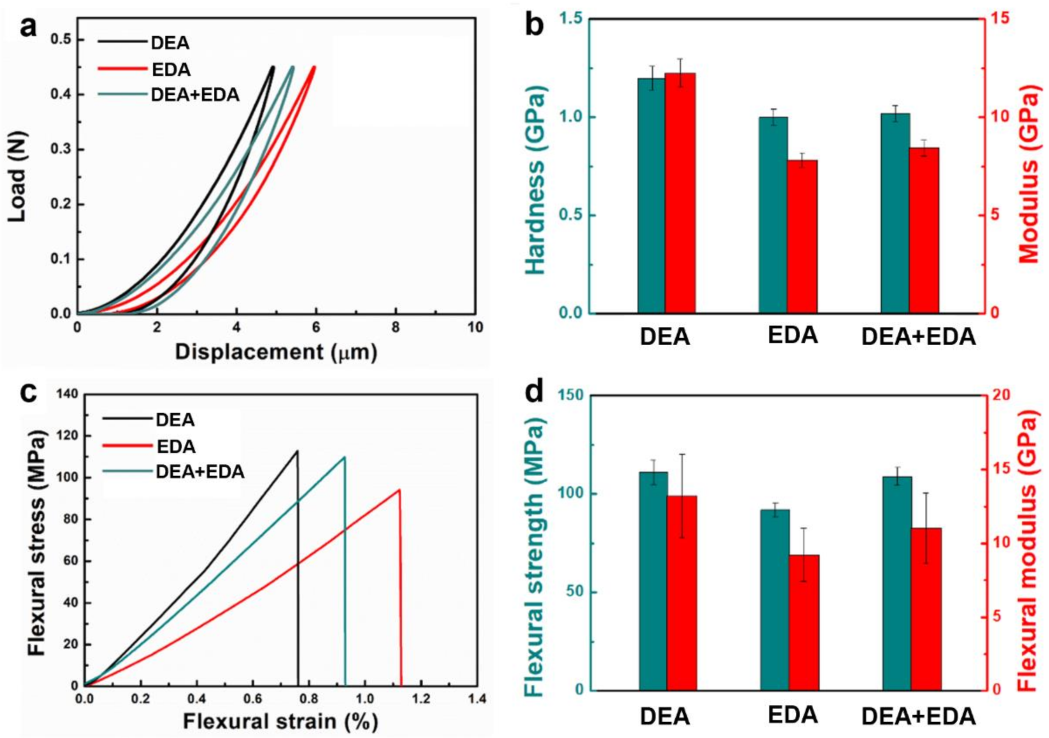

Figure S11. Mechanical properties of the nanocomposites containing DEA, EDA, and a mixture of DEA and EDA. (a-b) Nanoindentation tests: (a) load–displacement curves, and (b) hardness and modulus. (c-d) Three-point bending tests: (c) stress–strain curves, and (d) flexural strength and flexural modulus.

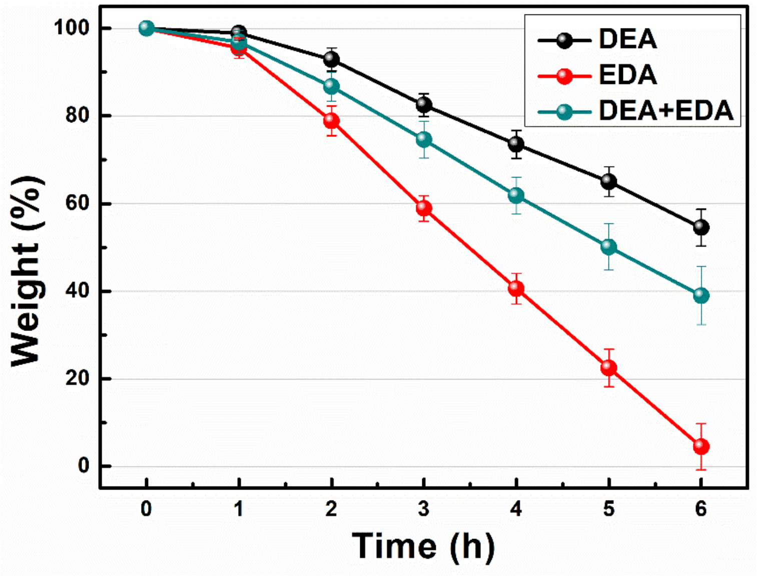

Figure S12. The weight change in the nanocomposites with different catalysts in water with time. The weight ratio of the nanocomposite/water is 20/500.

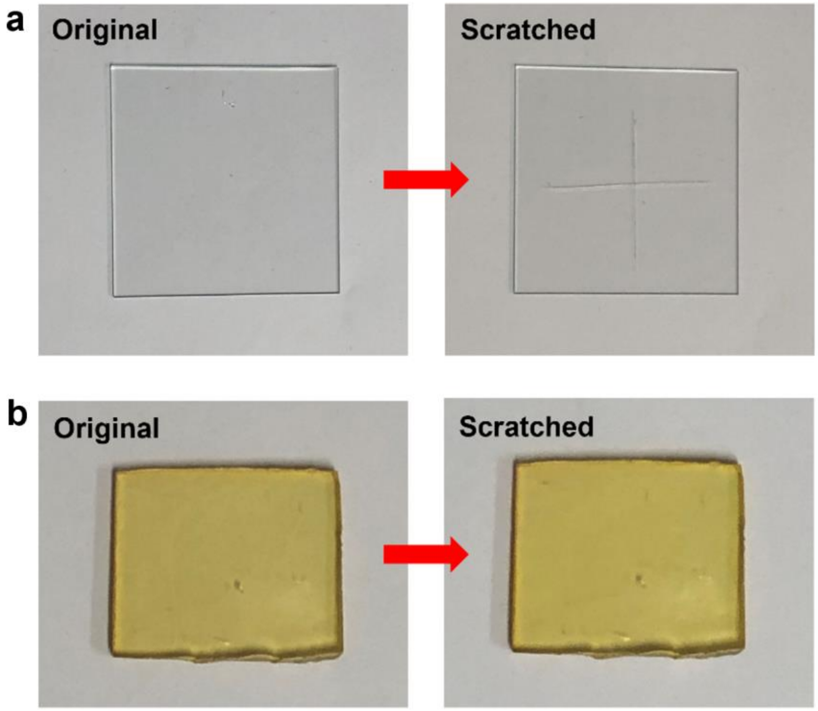

Figure S13. Photos showing that (a) the surface of the glass can be easily scratched by a glass cutter, while no scratch is formed on (b) the surface of our nanocomposite containing DEA and EDA by the cutter under the same force.

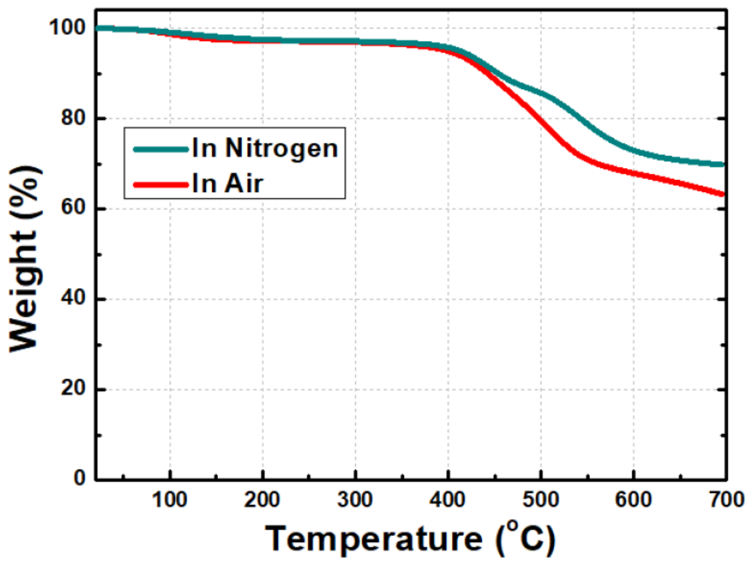

Figure S14. TGA curves of the nanocomposite containing DEA and EDA in air and nitrogen.

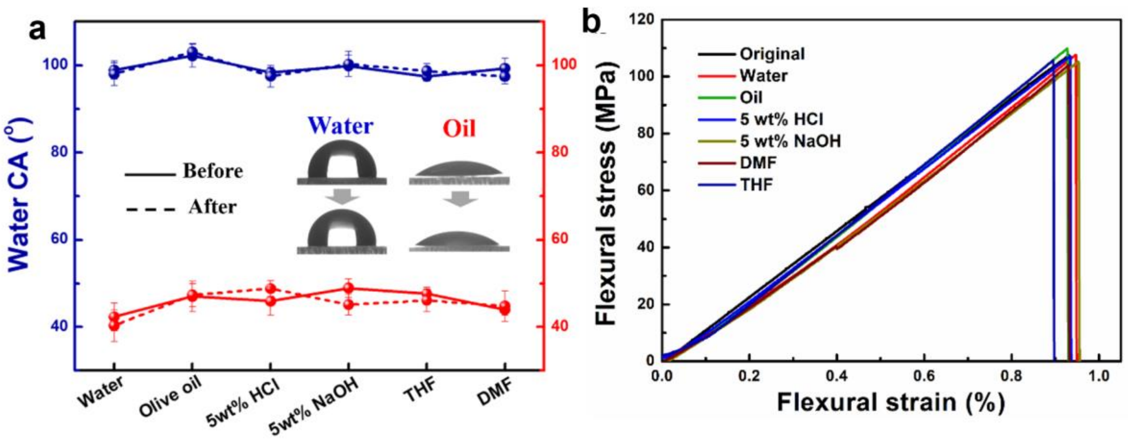

Figure S15. (a) Contact angles and (b) mechanical properties of the nanocomposite containing DEA and EDA before and after immersion in different solvents for 24 h.

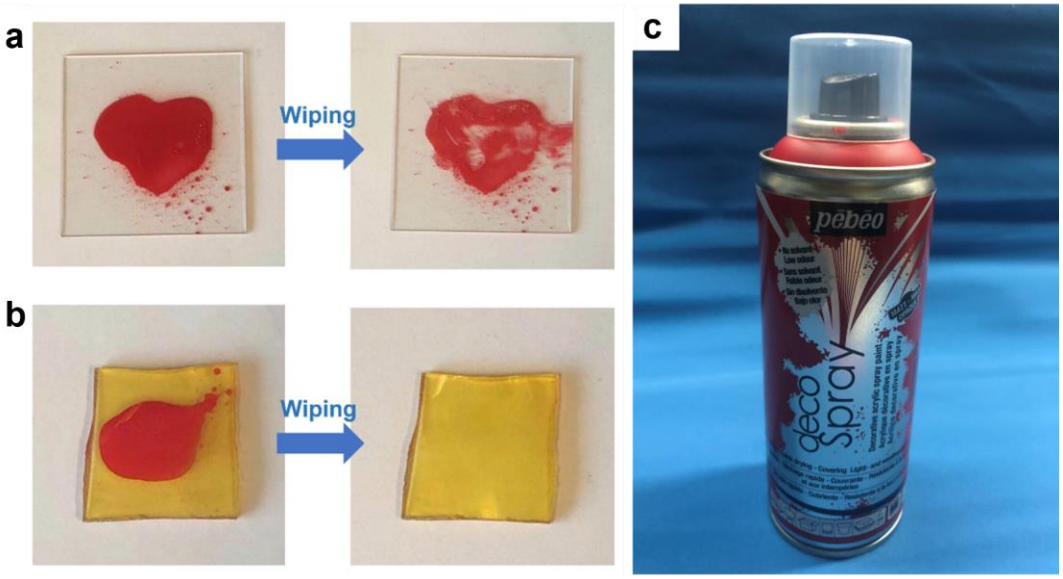

Figure S16. Photos of the polluted (a) glass and (b) nanocomposite containing DEA and EDA before and after wiping. (c) Photo of the water-based acrylic paint spray used as the pollutant.

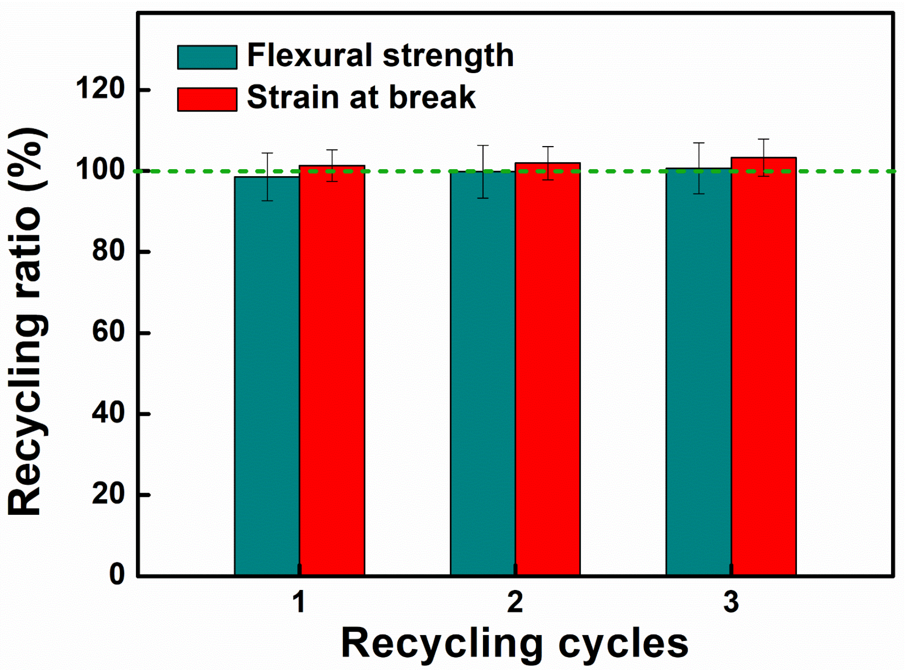

Figure S17. Recycling ratio of the nanocomposites after different recycling cycles.

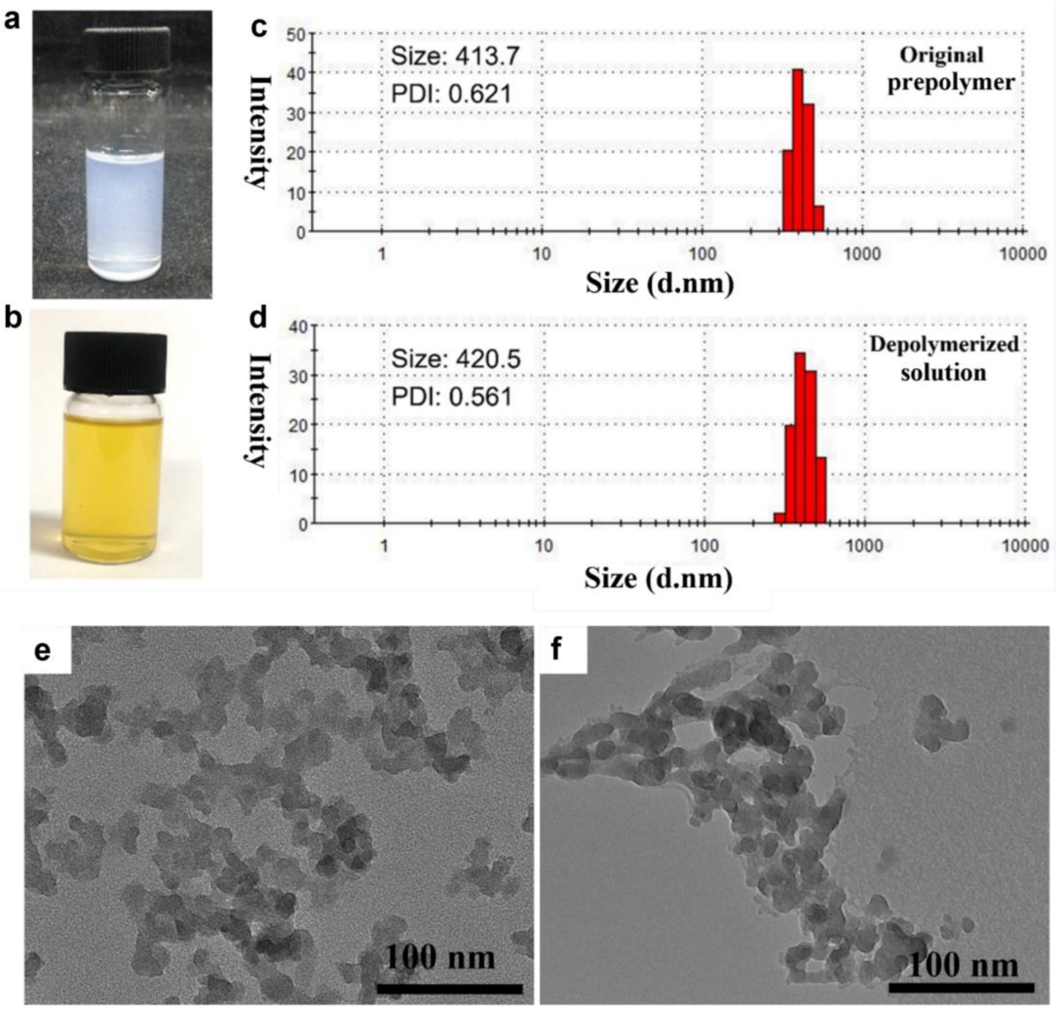

Figure S18. Photos of (a) the PTFPMS@APTES micelle dispersion and (b) the solution of depolymerized product in water. The size distributions of micelles in (c) the original prepolymer sol and (d) the depolymerized solution. TEM images of (e) the pristine PTFPMS@APTES micelles and (f) the micelles in the depolymerized solution.

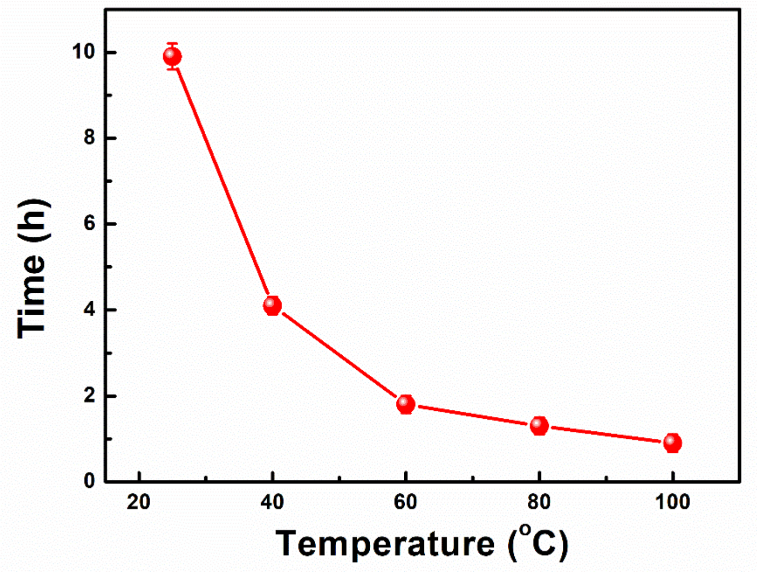

Figure S19. The impact of the temperature of water on the depolymerization time of the nanocomposite. The weight ratio of nanocomposite/water was 20/500, and the depolymerization time was judged by the complete disappearance of the nanocomposite containing DEA and EDA and the formation of a clear solution.

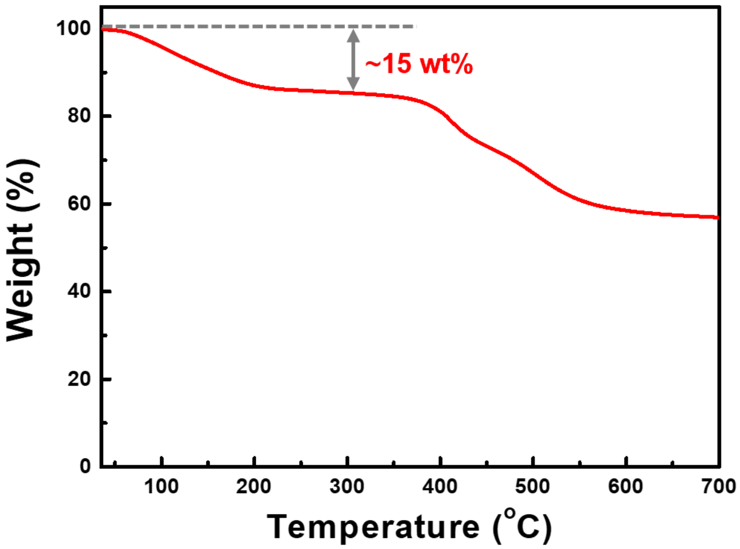

Figure S20. TGA curves of the solid prepolymer powder.

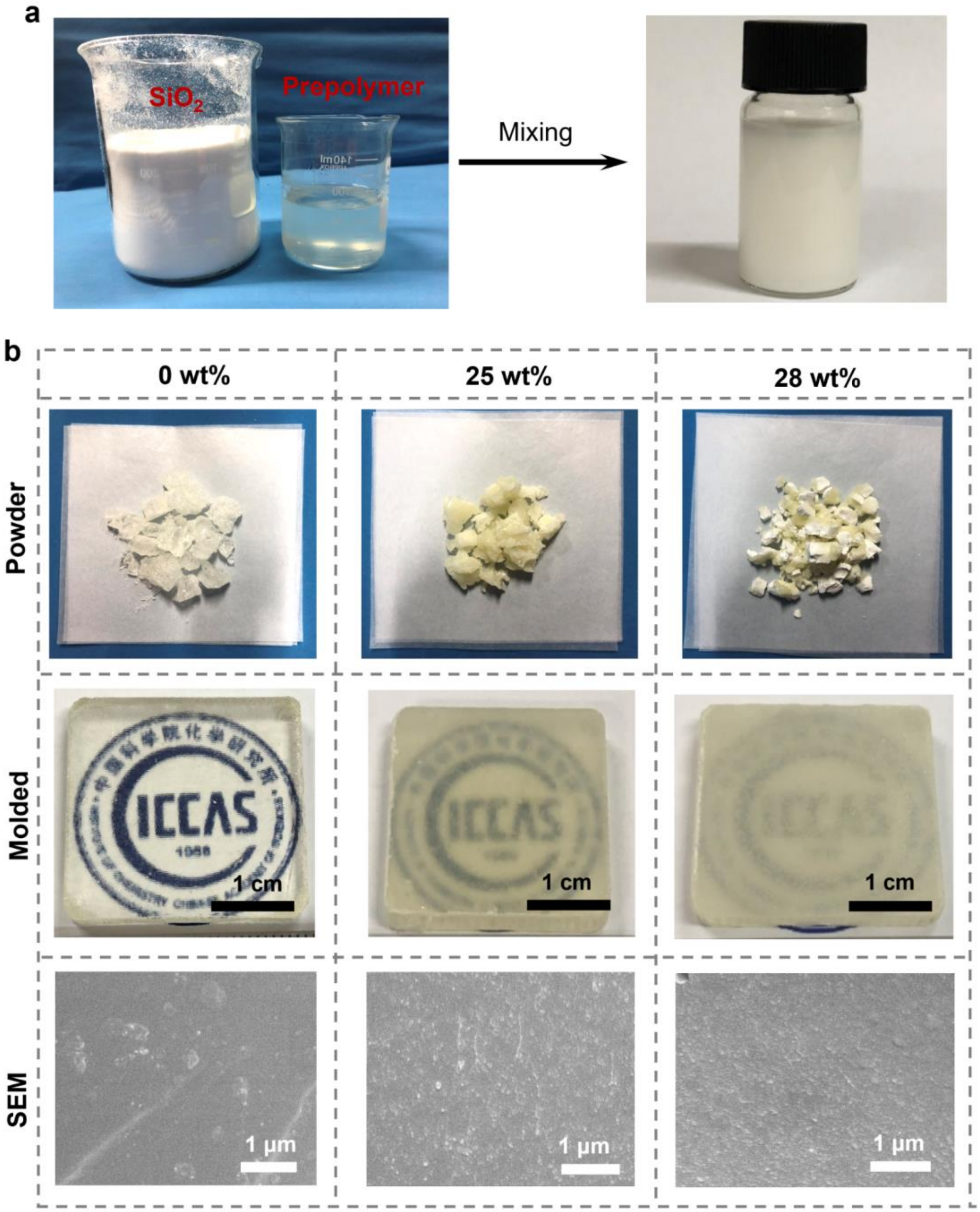

Figure S21. (a) Photos of SiO_2 nanoparticles mixed with the liquid prepolymer. (b) Photos of the composite powders, photos of molded nanocomposites, and SEM images of the nanocomposites with different SiO_2 content.

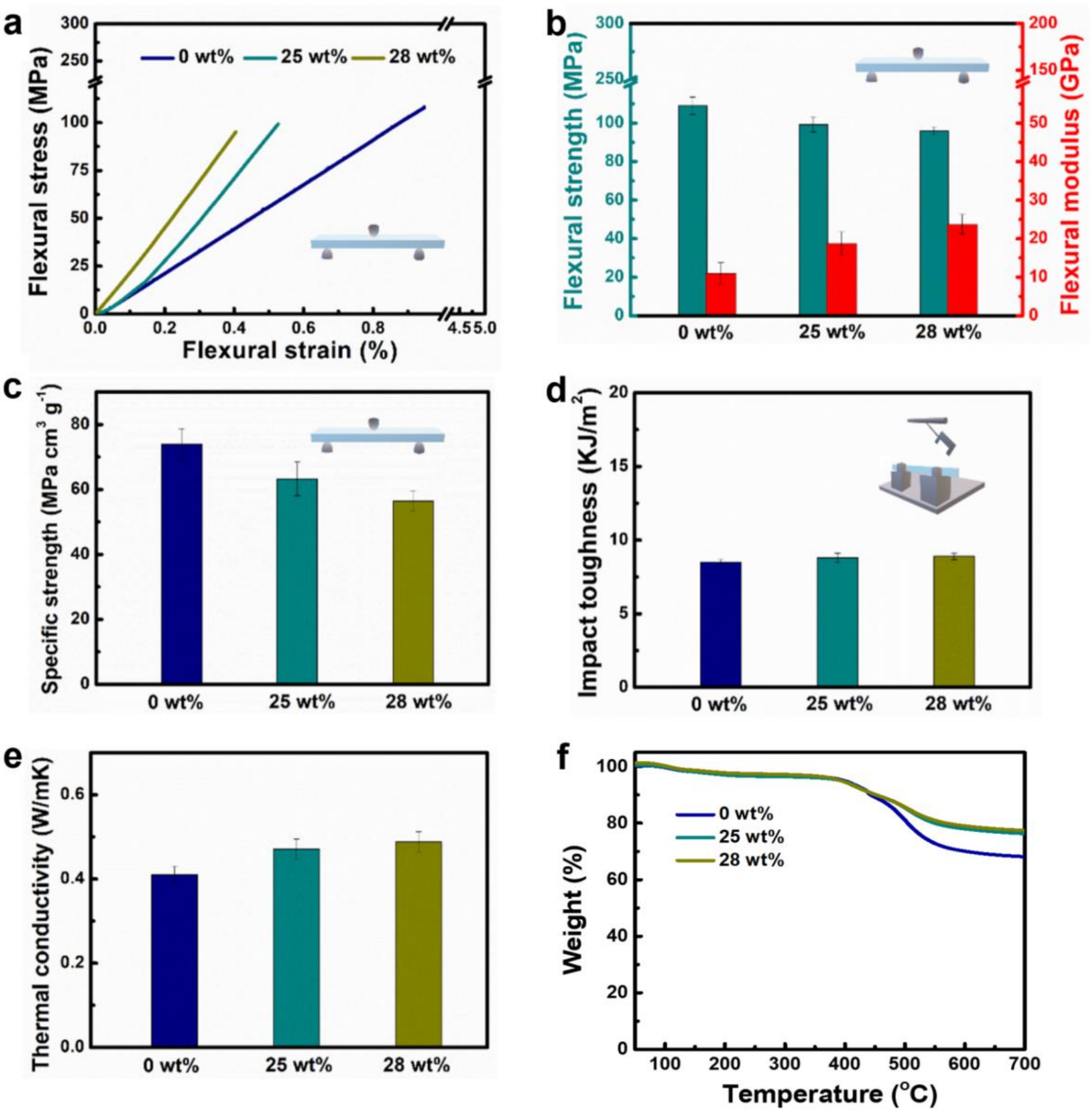

Figure S22. Material properties of the nanocomposites with 0, 25 and 28 wt% SiO_2 nanoparticles. (a-d) Mechanical properties of the nanocomposites: (a) flexural stress–strain curves, (b) flexural strength and flexural modulus, (c) specific flexural strength, (d) impact toughness. (e-f) Thermal properties of the nanocomposites: (e) thermal conductivity and (f) TGA curves showing the thermal stability.

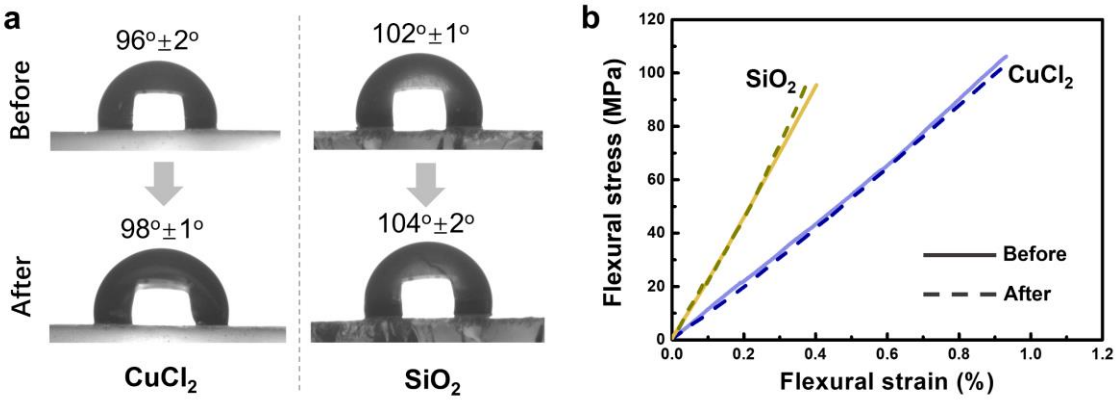

Figure S23. (a) Contact angles and (b) mechanical properties of the nanocomposites containing $CuCl_2$ and SiO_2 before and after immersion in water for 3 days.

Supplementary Tables

Table S1. Calculated degree of condensation (DC) from the ^{29}Si MAS NMR spectra of the hybrid Si-O-Si networks formed from the co-condensation of APTMS and TEOS with DEA or EDA as the catalyst.

Degree of condensation (DC)	DEA	EDA	DEA and EDA
DC_{APTMS}	97.6%	94.7%	97.0%
DC_{TEOS}	95.4%	93.4%	94.5%

Table S2. The chemical structural units of the prepolymers and the depolymerized products of the hybrid Si-O-Si networks formed from the co-condensation of APTMS and TEOS, as well as their molecular formulae, molecular weights, and symbols.

Chemical structural unit	Molecular formulae	Molecular weight	Symbol

**APTMS**	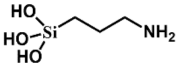	$C_3H_{11}NO_3Si$	137.1	
	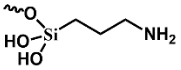	$C_3H_{10}NO_{2.5}SiR_1$	128.1	T_1
	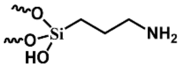	$C_3H_9NO_2SiR_2$	119.1	T_2
	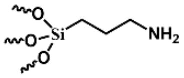	$C_3H_8NO_{1.5}SiR_3$	110.1	T_3
**TEOS**	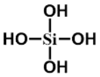	SiO_4H_4	96.1	
	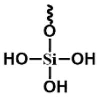	$R_1SiO_{3.5}H_3$	87.1	Q_1
	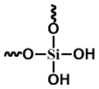	$R_2SiO_3H_2$	78.1	Q_2
	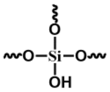	$R_3SiO_{2.5}H_1$	69.1	Q_3
	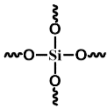	R_4SiO_2	60.1	Q_4
**DEA**	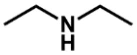	$C_4H_{11}N$	73.1	
**EDA**	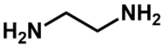	$C_2H_8N_2$	60.1	

Table S3. The molecular weights in MALDI-TOF-MS spectra of the prepolymers and the depolymerized products (Figure 2h, Figure S4) of the hybrid Si-O-Si networks formed from the co-condensation of APTMS and TEOS with DEA or EDA as the catalyst, and their possible structural units.

Catalyst	State	Molecular weights (possible structural units)
**DEA**	Prepolymer	410(T_2+2T_3+DEA); 428(T_1+T_2+T_3+DEA); 440(4T_3); 449(2T_1+T_2+DEA); 528(2T_2+2T_3+DEA, 2T_1+T_2+Q_2+DEA)
	Depolymerized product	508(T_1+T_2+T_3+Q_2+DEA, 2T_1+T_2+Q_4+DEA); 528(2T_2+2T_3+DEA, 2T_1+T_2+Q_2+DEA); 549(2T_1+2T_3+DEA); 572(3T_1+T_2+Q_3, 3T_1+T_3+Q_2, 4T_1+Q_4); 636(3T_1+T_2+T_3+Na, 2T_1+3T_2+Na, T_2+4T_3+Q_2, T_1+4T_3+ Q_3)
**EDA**	Prepolymer	398(2T_1+T_2+Na, T_2+2T_3+EDA, T_2+2T_3+Q_4, 3T_3+Q_3); 417(T_2+2T_3+Q_2); 440(4T_3, 3T_2+Q_4+Na, 3T_2+EDA+Na, 2T_2+T_3+Q_3+Na); 518(2T_2+2T_3+Q_4, 2T_2+2T_3+EDA)
	Depolymerized product	554(2T_1+T_2+T_3+Q_3, 3T_1+T_3+Q_4, 4T_2+Q_2, 3T_2+T_3+Q_1); 658(6T_3, T_2+3T_3+3Q_3, 2T_2+2T_3+2Q_3+EDA, 2T_2+2T_3+Q_2+2Q_4, T_2+3T_3+Q_2+Q_3+Q_4, 2T_2+2T_3+Q_2+Q_4+EDA); 686(3T_1+T_2+T_3+DEA, T_2+3T_3+Q_1+Q_2+DEA); 720(6T_3+Q_4, 6T_3+EDA, T_1+T_2+3T_3+2Q_4+Na, 3T_2+2T_3+2Q_4+Na)
**DEA and EDA**	Prepolymer	417(T_1+2T_3+Q_3, T_1+T_2+T_3+Q_4, 3T_2+Q_4, T_2+2T_3+Q_2, 3T_2+EDA, T_1+T_2+T_3+EDA); 440(4T_3, T_1+T_2+T_3+EDA+Na, 3T_2+EDA+Na, T_1+T_2+T_3+Q_4+Na, 3T_2+Q_4+Na); 448(2T_1+T_2+DEA)
	Depolymerized product	508(T_1+T_2+T_3+Q_2+DEA, 2T_1+T_2+Q_4+DEA); 572(3T_1+T_2+Q_3, 3T_1+T_3+Q_2, 4T_1+Q_4, 4T_1+EDA); 595(5T_2, 2T_1+T_2+T_3+Q_1+Na, T_2+3T_3+Q_1+EDA, T_2+3T_3+Q_1+Q_4, 4T_3+Q_1+Q_3, 2T_1+2T_2+Q_2+Na, 3T_1+T_3+Q_2+Na, 4T_3+2Q_2); 658(6T_3, T_2+3T_3+3Q_3, 2T_2+2T_3+2Q_3+EDA, 2T_2+2T_3+Q_2+2Q_4, T_2+3T_3+Q_2+Q_3+Q_4, 2T_2+2T_3+Q_2+Q_4+EDA); 686(3T_1+T_2+T_3+DEA, T_2+3T_3+Q_1+Q_2+DEA)

Table S4. Density and mechanical properties from nanoindentation tests of our nanocomposites and some common materials.

Materials	Density (g/cm^3)	Hardness (GPa)	Modulus (GPa)	H/E (%)	W_e (%)
**Nanocomposite**	1.36±0.04	1.02±0.07	8.44±0.41	12.1±0.4	79.8±0.4
**Nanocomposite with 25 wt% nanofillers**	1.57±0.04	1.07±0.06	9.84±0.27	10.8±0.2	71.2±0.5
**Nanocomposite with 28 wt% nanofillers**	1.48±0.06	1.13±0.03	8.61±0.16	13.2±0.1	80.0±1.0
**PC**	1.18~1.22	0.22	4.36	5.1	35.8
**PMMA**	1.17~1.20	0.29	5.49	5.4	39.9
**PP**	0.89~0.93	0.19	3.42	5.7	38.3
**ABS**	1.04~1.08	0.14	2.62	5.1	35.0
**Al alloy**	2.5~2.9	0.49	95.48	0.5	3.5
**Cu**	8.96	1.24	113.3	1.1	7.9
**ZrO_2**	5.85	10.18	223.37	6.3	49.5
**Al_2O_3**	3.92	12.46	392.64	3.5	40.7
**Glass**	2.2~2.5	7.47	74.84	10.0	56.4
